# The role of choline-based ionic liquids in modulating the thermophysical properties of *d-*fructose solutions

**DOI:** 10.1186/s13065-025-01491-5

**Published:** 2025-06-05

**Authors:** Sara Dorosti, Hemayat Shekaari, Mohammad Bagheri, Fariba Ghaffari, Masumeh Mokhtarpour

**Affiliations:** Department of Physical Chemistry, Faculty of Chemistry, University of Tabriz, Tabriz, Iran

**Keywords:** Choline based*-*ionic liquids, *d-*Fructose, Thermophysical properties, Taste behavior, DFT-COSMO calculations

## Abstract

**Abstract:**

In order to better understand how choline-based ionic liquids can improve the process of converting sugar to bioethanol, our study examined how *d-*fructose interacted with aqueous solutions of choline salicylate ([Ch][Sal]), choline formate ([Ch][For]), and choline acetate ([Ch][Ace]). A series of measurements including density, speed of sound, viscosity, and electrical conductivity were performed across varying temperatures and concentrations to assess the physicochemical performance of *d-*fructose in the studied solutions. The obtained properties including apparent molar volume (*V*_φ_), apparent molar isentropic compressibility (*κ*_φ_), viscosity *B*-coefficients, and molar conductivity (*Λ*) were analyzed to gain insights into the nature of intermolecular interactions. The calculated standard partial molar volume (*V*_φ_^0^) of *d-*fructose indicated enhanced interactions between *d-*fructose and the ionic liquids. Hepler’s constant values pointed to a structure-making tendency of *d-*fructose, particularly in aqueous [Ch][Sal] solutions. To further probe these interactions, DFT-COSMO calculation was employed, revealing that [Ch][Sal] exhibits preferentially the most energetically favorable interactions. Additionally, values of apparent specific volume (*ASV*) and apparent specific isentropic compressibility (*ASIC*) suggested that the ILs have a negligible influence on the inherent physical characteristics of *d-*fructose. As the temprature increased, the hydration number of *d-*fructose decreased, which can be due to the weakening of hydrogen bonding with water. These results highlight [Ch][Sal] ionic liquid as a promising medium for potentially promoting sugar-to-bioethanol conversion.

**Graphical Abstract:**

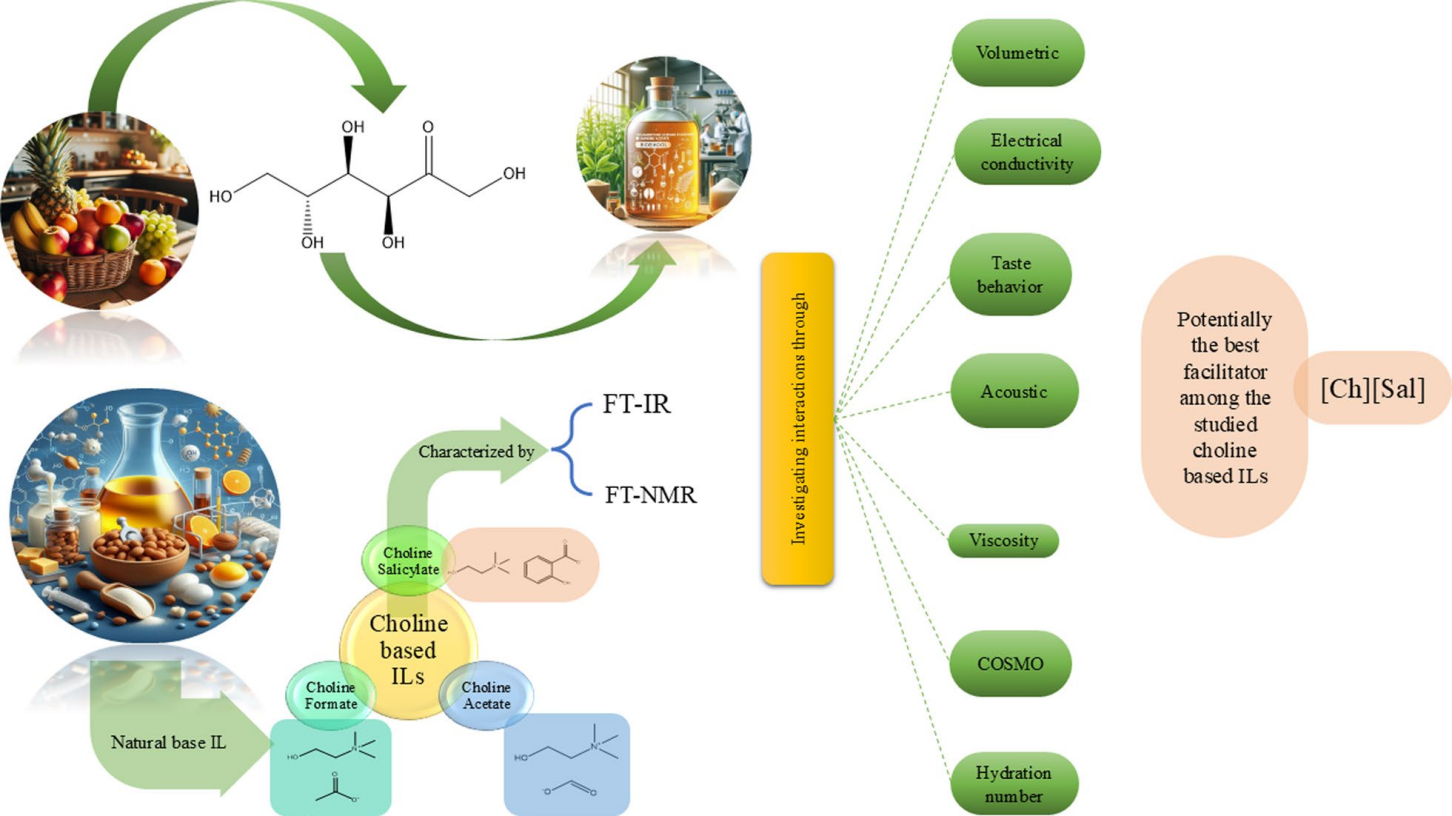

**Supplementary Information:**

The online version contains supplementary material available at 10.1186/s13065-025-01491-5.

## Introduction

As fossil fuel reserves dwindle, the urgency to explore renewable alternatives becomes increasingly apparent. In response to these pressing challenges, researchers have turned their attention to bio-based energy sources, such as bioethanol. One of the most efficient methods for producing bioethanol involves the conversion of sugars [1]. *d-*fructose is the sweetest naturally occurring sugar that is found in many fruits and honey. Aqueous solutions of sugars have been extensively studied due to their scientific significance, practical applications, and their potential as inexpensive and recyclable resources in various chemical, pharmaceutical, biological, and food related fields. However, sugars limited solubility presents a significant challenge in this context. While certain solvents like DMF and DMSO can dissolve sugars, their application is limited due to their ability to inactivate enzymes in esterification reactions. Traditionally, organic solvents have been employed for the facilitation of the sugar conversion rate into bioethanol. However, the use of organic solvents raises concerns regarding the environmental impact and sustainability [2].

The presence of ionic liquids in aqueous sugar solutions creates a new type of ternary system with broad applications in diverse industries. Therefore, understanding the interaction interplay between sugars and ionic liquids is of great importance for the design and optimization of processes involving these systems. Thermodynamic studies are crucial for elucidating the intricate interactions between sugars and ionic liquids. Such investigations can provide valuable insights into the underlying forces driving these interactions [3–6].

Choline-based ILs are a class of ionic liquids derived from the choline cation, a quaternary ammonium compound containing a hydroxyl group (HO-CH2 CH2 N(CH3)3+). Choline is a naturally occurring compound found in living organisms and has been recognized as an essential nutrient, vitamin B4. Owing to their unique properties, such as low toxicity, biodegradability, and diverse applications, choline-based ILs have attracted considerable interest in recent years. Choline-based ILs, have been known for their low toxicity and biodegradability, which makes them an environmentally friendly approach to enhance sugar solubility and conversion rates, addressing the limitations associated with traditional organic solvents [7, 8].

The selection of appropriate ionic liquids (ILs) for a given task is crucial, particularly considering their environmental impact. Choline-based ILs, classified as third*-*generation and aprotic ILs, are among the greenest options. The choline cation, a vital nutrient involved in various bodily functions, including brain health and acetylcholine production, underscores the potential biocompatibility of these ILs. Assessing the potential of choline-based ionic liquids for enhancing sugar-to-bioethanol conversion requires a detailed understanding of their intermolecular interactions with sugars [9]. Choline-based ionic liquids (ILs) and deep eutectic solvents (DESs) have demonstrated significant potential in facilitating bioethanol production from lignocellulosic biomass. These solvents efficiently interrupt the recalcitrant structure of biomass, thus improving sugar yields and enhancing lignin recovery [10]. Choline chloride-based deep eutectic solvents (DESs), particularly in combination with glycerol, have exhibited significant pretreatment efficiency, yielding over 80% sugar following enzymatic hydrolysis [11].

This research aimed to elucidate the impact of choline-based ionic liquids (ILs) on the volumetric, acoustic, and transport properties of aqueous *d-*fructose solutions. To achieve this, a series of experiments were conducted to explore the interactions concerning *d-*fructose and the specific studied choline-based ILs: choline formate ([Ch][For]), choline acetate ([Ch][Ace]), and choline salicylate ([Ch][Sal]). These experiments were carried out at varying concentrations of the ILs and over a temperature range of (298 to 318) K. By investigating the density (*ρ*), speed of sound (*u*), electrical conductivity, and viscosity (*η*), of the solutions, their thermophysical properties were derived which helped in achieving an understanding of the molecular interactions concerning the choline based ionic liquids, and *d-*fructose in aqueous media. The prepared aqueous *d-*fructose solutions with different concentrations ranges (0.0000 to 0.0900) mol·kg⁻^1^ and temperatures spanning from (298 to 318) K were investigated. Furthermore, the electrical conductivity of aqueous solutions containing choline-based ILs and *d-*fructose was studied at a steady temperature of 298 K and within the same concentration range. As a result of the taken measurements the molar related properties of apparent molar volume, $V_{\varphi }$, standard partial molar volume, $V_{\varphi }^{0}$, limiting molar conductivity, $\Lambda_{0}$, viscosity *B*-coefficients has been computed. The COSMO calculations were utilized to extract key parameters including the sigma profile (*σ*-profile), surface cavity area (*A*), total volume of cavity (*V*), dielectric solvation energy, and the energy levels of the highest occupied and lowest unoccupied molecular orbitals (HOMO–LUMO).

## Experimental measurements

### Materials

The sources, CAS numbers, and purity levels of the compounds used in this investigation are all listed in detail in Table 1. The water used in the trials was deionized and double distilled, and its specific conductivity was less than 1 μS·cm⁻^1^.

**Table 1:** Descriptions of the used chemicals

Chemical name	Chemical formula	Provenance	CAS.no	Molar mass (g∙mol^−1^)	Mass fraction (purity)
*d-*Fructose	C_6_H_12_O_6_	Merck	50–99-7	180.16	> 99%
Choline chloride	C_5_H_14_ClNO	Merck	67-48-1	139.62	> 98%
Salicylic acid	C_7_H_6_O_3_	Merck	69–72-7	138.12	> 99%
Formic acid	CH_2_O_2_	Merck	64-18-6	46.03	> 99%
Acetic acid	C_2_H_4_O_2_	Merck	64-19-7	60.050	> 99%
Dichloromethane (DCM)	C_4_H_11_NO_3_	Merck	75-09-2	84.93	> 99%
Choline salicylate	C_12_H_19_NO_4_	Synthesized	2016-36-6	241.29	> 98%
Choline formate	C_6_H_15_NO_3_	Synthesized	9031-54-3	149.19	> 98%
Choline acetate	C_7_H_17_NO_3_	Synthesized	14586-35-7	163.22	> 98%

### Preparation and characterization of the choline- based ILs through ^1^H-NMR & FT-IR analysis

The synthesis routes for choline salicylate ([Ch][Sal]), choline formate ([Ch][For]), and choline acetate ([Ch][Ace]) are detailed in Figures S1-S3. The corresponding FT-IR (Figures S4-S6) and ^1^H-NMR (Figures S7-S9) spectra, along with their interpretations, have been provided in the Supplementary Information.

### Density and speed of sound measurements

A Shimadzu-AW220 analytical balance with a precision of ± 2 × 10⁻^4^ kg was utilized for preparing each solution. A DSA 5000 digital densimeter (Anton Paar, Austria) has been utilized to measure the density (*ρ*) and speed of sound (*u*) of binary mixtures of [Ch][Sal], [Ch][For], and [Ch][Ace] in water, along with ternary systems containing *d-*fructose in aqueous choline based ILs solutions. This apparatus has a Peltier thermostat to provide steady temperature conditions and a high-precision vibrating tube that runs at about 3 MHz. The manufacturer’s air/water protocol was followed for calibrating the densimeter. Calibration involved the use of standard solutions—specifically, distilled water with conductivity below 1 μS·cm⁻^1^ whose density at a controlled temperature was compared to reference values, and the device settings were fine-tuned to reduce any measurement deviation. The estimated uncertainties for density and speed of sound were around 0.06 × 10⁻^3^ g·cm⁻^3^ and 1 m·s⁻^1^, respectively.

### Viscosity measurement

An Austrian rolling-ball viscometer, the Anton Paar Lovis 2000 M/ME, was used to measure viscosity. The apparatus features an integrated thermostat based on Peltier technology, ensuring precise temperature control within ± 0.05 K. This apparatus works on the falling ball method, which measures the time it takes for a steel ball to descend through a sample-filled, pre-calibrated glass capillary. Using the measured fall time and associated density values, the kinematic and dynamic viscosities were computed. Using approved viscosity standards, the company carried out the calibration. The calculated overall measurement uncertainty for viscosity was 0.001 mPa·s.

### Electrical conductivity measurements

The Metrohm 712 conductivitymeter equipped with a dipped platinized electrode cell has been utilized for measuring the electrical conductivity (cell constant: 0.880 cm⁻^1^). By using an aqueous solution of 0.01 mol·kg⁻^1^ KCl, the cell constant was obtained. Each measurement involved filling the cell with a precisely weighed volume of degassed, double distilled, deionized water with a known amount of *d-*fructose. A certain volume of pure ionic liquid (IL) was then added to the mixture, which was constantly swirled ensuring uniformity. A Julabo ED thermostat and a circulating water bath were used to keep the temperature within ± 0.2 K. The specific conductivity measurements were calculated to have an error of less than 0.5%.

## Results and discussion

### Theoretical framework

**Fig. 1:**
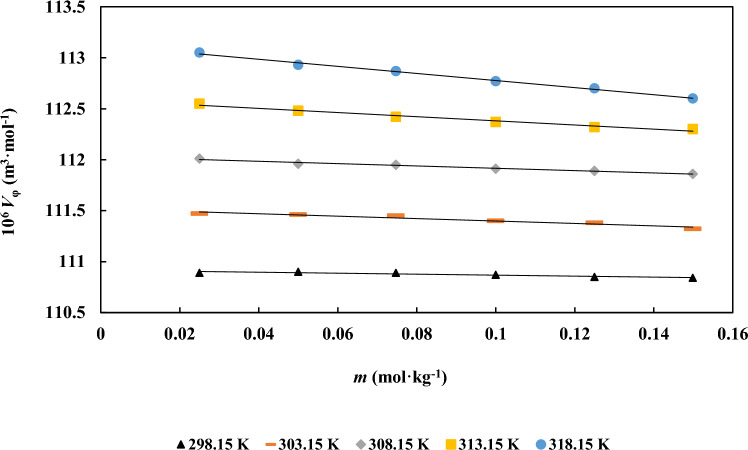
The optimized molecular structure and *σ*-profile of **a** *d-*fructose, **b** [Ch][For], **c** [Ch][Ace], **d** [Ch][Sal] and **e** sigma profile plot from Dmol^3^ and COSMO result

The theoretical approach of this work is based on Density Functional Theory (DFT) using the Dmol^3^ module in the Materials Studio package (Biovia, 2023). To balance computational accuracy and efficiency, the Generalized Gradient Approximation (GGA) with the VWN-BP functional recommended by the Dmol^3^ developers was applied. For COSMO calculations, water was chosen as the solvent model. A two-step computational approach was adopted. Initially, geometry optimization was performed using the GGA VWN-BP functional and the DND (3.5) basis set. Subsequently, COSMO calculations were carried out to incorporate the effects of solvation. In Fig. 1, the *σ*-profiles produced by COSMO are shown, and in Table 2, the calculated dielectric solvation energies, surface cavity area (*A*), surface cavity volume (*V*), HOMO and LUMO values, and their corresponding energies for *d-*fructose and the choline-based ILs are recorded.

**Table 2:** The surface area (*A*) and total volume of cavity (*V*), dielectric (solvation) energy, HOMO and LUMO values and energies obtained from COSMO and Dmol^3^ calculations

Material	A (Å^2^)	V (Å^3^)	Dielectric (solvation) energy (kcal·mol^−1^)	HOMO	LUMO	*E*_HOMO_ ev	*E*_LUMO_ ev
*d-*Fructose	243.064	253.151	− 19.50	57	58	− 6.194	− 2.199
[Ch][Sal]	283.071	286.072	− 82.19	65	66	− 4.365	− 1.149
[Ch][Ace]	226.287	210.814	− 84.45	45	46	− 4.439	0.327
[Ch][For]	205.791	191.752	− 65.24	41	42	− 4.737	0.348

In this research, choline-based ILs display consistent patterns as the length of the alkyl chain increases, as indicated in Table 2. From [Ch][For] to [Ch][Ace] to [Ch][Sal], the dielectric solvation energy gets progressively lower, suggesting stronger IL–water interactions with longer alkyl chains. At the same time, the energy of highest occupied orbital molecule and lowest unoccupied orbital molecule shift upward (i.e., become less negative), reflecting a diminished ability for electron donation and acceptance. As the alkyl chain lengthens, *E*_LUMO_ values show an interestingly more complex pattern, changing from positive to negative. Overall, these results highlight that increasing the chain length notably affects both the electronic structure and solvation behavior of the ILs. The more negative dielectric solvation energy indicates enhanced interaction with the solvent, while the observed shifts in frontier orbital energies suggest changes in the ILs’ electron transfer characteristics. The computed surface cavity volumes for the studied substances are presented in Table 2. Notably, [Ch][Sal] exhibits the highest surface cavity volume among the investigated ILs. This suggests that [Ch][Sal] has the most favorable theoretical interactions with water, potentially facilitating the conversion of *d-*fructose. The surface cavity volume indicates the interactions between the solute and the solvent. The higher values of the surface cavity volume indicates stronger interactions. 

### Volumetric results

The density (*ρ*) measurements for *d-*fructose in pure water and in aqueous solutions containing 0.0500, 0.1000, and 0.1500 mol∙kg⁻^1^ of the three choline-based ionic liquids [Ch][Sal], [Ch][For], and [Ch][Ace] are shown in Table S1. Analysis of these data unveils various notable insight. Firstly, an increment in the density with rising *d-*fructose content, which aligns with the higher intrinsic density of *d-*fructose related to water. In proportion to the amount of *d-*fructose added, the solution's total density increases. Moreover, ILs with longer alkyl chains tend to yield higher solution densities. For instance, [Ch][Sal], having the longest chain among the studied ILs, shows a higher density relative to [Ch][For] and [Ch][Ace]. This can be explained by stronger van der Waals forces that promote tighter molecular packing. Lastly, temperature and IL concentration exert opposite effects: density generally decreases with increasing temperature due to solvent expansion, whereas greater IL content typically raises the density, given that ionic liquids are denser than water. The apparent molar volumes ($V_{\varphi }$) of *d-*fructose in these systems were calculated using the following equation [12]:

1 $$V_{\varphi } = \frac{M}{\rho } - \frac{{(\rho - \rho_{0} )}}{{m\rho \rho_{0} }}$$

**Fig. 2:**
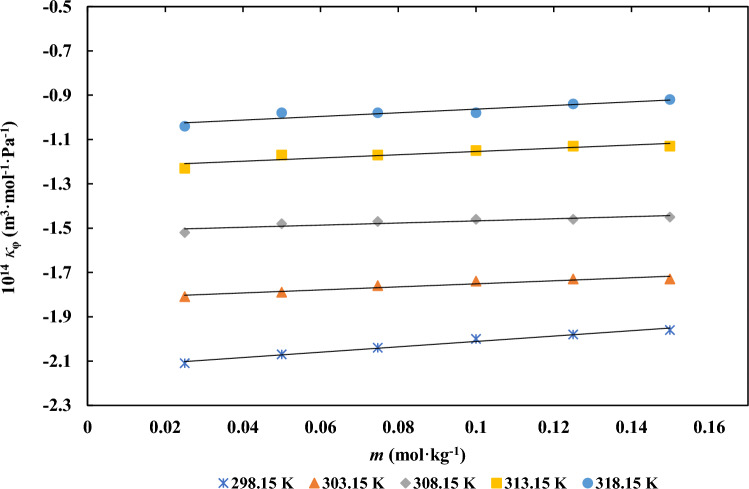
Apparent molar volumes ($V_{\varphi }$/m^3^.mol^−1^) of *d-*fructose versus its molality m (mol.kg^−1^) in aqueous [Ch][Ace] solutions: (**a**) Different [Ch][Ace] molalities ■, 0.0900; ◆, 0.0600; ▲, 0.0300 at *T* = 298 K and (**b**) A fixed [Ch][Ace] molality of 0.0300 mol kg^−1^ at varied temperatures

In this equation, *M* denotes the molar mass of *d-*fructose, *m* is its molality in aqueous ionic liquid solutions, while $\rho$, and $\rho_{0}$ represent the densities of the *d-*fructose in aqueous ILs solution and _*D*__-_fructose in water systems, respectively. Table S1 also includes the calculated apparent molar volume ($V_{\varphi }$) values for *d-*fructose in both pure water and aqueous IL solutions across temperatures ranging from 298.15 to 318.15 K, in 5 K increments. Figure 2 illustrates the variation of $V_{\varphi }$ for *d-*fructose in aqueous [Ch][Ace] solutions. The results show that $V_{\varphi }$ increases consistently with IL concentration over the examined temperature range. A strong linear relationship was found between $V_{\varphi }$ and *d-*fructose molality, and similar trends were observed for the other IL systems. Consequently, standard partial molar volumes ($V_{\varphi }^{0}$) at infinite dilution were derived by applying least-squares regression based on Masson’s equation [12]:

2 $$V_{\varphi } = V_{\varphi }^{0} + S_{v} m$$

where, S_v_ stands for the empirical parameters. At infinite dilution, solute–solvent interactions become insignificant, hence *V*_φ_^0^ offer important information about these interactions. Table 3 reports the values of *S*_v_ together with the standards deviation of those values. It is significant that every number, which represents solute–solvent interactions, is positive and shows an upward trend as the temperature and content of ionic liquids increase. Increased solute–solvent interactions and decreased water electrostriction are the causes of this phenomenon. It is most likely the release of solvent molecules into the bulk that causes the amplification that is seen at higher temperatures. Likewise, the higher values found for ternary systems point to a similar occurrence. values, the following formula can be used to express temperature dependency [13]:

3 $$V_{\varphi }^{0} = A + BT + CT^{2}$$

**Table 3:** The standard partial molar volumes ($V_{\varphi }^{0}$), adjustable parameters of Eq. 2 ($S_{v}$ $.$), transfer volumes ($\Delta_{tr} V_{\varphi }^{0}$), and related standard deviations ($\sigma ( V_{\varphi }^{0} )$) for *d-*fructose in aqueous solutions of ILs at different temperatures^a^

*T* (K)	10^6^ *S*_v_ (m^3^·kg·mol^−2^)	10^6^ *V*_φ_^0^ (m^3^·mol^−1^)	Δ_tr_*V*_φ_^0^ (cm^3^·mol^−1^)	*σ* (V^0^_φ_)
*d-*Fructose + water				
298.15	8.82 ± 0.220	109.46 ± 0.021	–	0.02
303.15	8.64 ± 0.426	109.95 ± 0.041	–	0.04
308.15	8.86 ± 0.691	110.45 ± 0.067	–	0.06
313.15	9.00 ± 0.375	110.94 ± 0.037	–	0.03
318.15	9.35 ± 0.384	111.35 ± 0.037	–	0.03
*d-*Fructose in aqueous solutions of [Ch][Sal] (0.0299 mol.kg^−1^)				
298.15	8.62 ± 0.693	109.59 ± 0.067	0.13	0.06
303.15	9.33 ± 0.889	110.17 ± 0.086	0.23	0.07
308.15	9.48 ± 0.607	110.78 ± 0.058	0.33	0.05
313.15	9.45 ± 0.354	111.45 ± 0.034	0.51	0.03
318.15	8.97 ± 0.480	112.09 ± 0.046	0.75	0.04
*d-*Fructose in aqueous solutions of [Ch][Sal] (0.0599 mol.kg^−1^)				
298.15	2.41 ± 0.159	110.72 ± 0.015	1.26	0.01
303.15	2.93 ± 0.237	111.15 ± 0.023	1.20	0.02
308.15	4.22 ± 0.309	111.48 ± 0.030	1.03	0.03
313.15	7.32 ± 0.262	111.85 ± 0.025	0.90	0.02
318.15	8.20 ± 0.816	112.29 ± 0.079	0.94	0.07
*d-*Fructose in aqueous solutions of [Ch][Sal] (0.0898 mol.kg^−1^)				
298.15	2.85 ± 0.166	110.84 ± 0.016	1.38	0.01
303.15	3.58 ± 0.186	111.20 ± 0.018	1.25	0.02
308.15	4.51 ± 0.346	111.62 ± 0.034	1.17	0.03
313.15	6.52 ± 0.313	112.04 ± 0.030	1.10	0.03
318.15	7.59 ± 0.352	112.48 ± 0.034	1.13	0.03
*d-*Fructose in aqueous solutions of [Ch][For] (0.0299 mol.kg^−1^)				
298.15	2.415 ± 0.400	110.464 ± 0.039	1.033	0.01
303.15	2.497 ± 0.277	110.966 ± 0.027	1.016	0.02
308.15	2.592 ± 0.277	111.432 ± 0.027	0.982	0.02
313.15	2.779 ± 0.413	111.825 ± 0.040	0.885	0.03
318.15	2.305 ± 0.274	112.252 ± 0.027	0.902	0.02
*d-*Fructose in aqueous solutions of [Ch][For] (0.0598 mol.kg^−1^)				
298.15	2.009 ± 0.363	110.655 ± 0.035	1.249	0.04
303.15	0.813 ± 0.671	111.153 ± 0.065	1.203	0.06
308.15	0.881 ± 0.659	111.535 ± 0.064	1.085	0.06
313.15	0.854 ± 0.337	111.937 ± 0.033	0.997	0.03
318.15	0.763 ± 0.544	112.354 ± 0.053	1.004	0.05
*d-*Fructose in aqueous solutions of [Ch][For] (0.0898 mol.kg^−1^)				
298.15	2.396 ± 0.291	110.845 ± 0.028	1.422	0.02
303.15	2.154 ± 0.395	111.281 ± 0.038	1.331	0.03
308.15	2.224 ± 0.987	111.653 ± 0.096	1.203	0.08
313.15	2.122 ± 0.463	112.032 ± 0.045	1.092	0.04
318.15	1.605 ± 0.244	112.458 ± 0.024	1.108	0.02
*d-*Fructose in aqueous solutions of [Ch][Ace] (0.0299 mol.kg^−1^)				
298.15	− 0.532 ± 0.090	110.919 ± 0.008	1.463	0.01
303.15	− 1.171 ± 0.153	111.515 ± 0.015	1.568	0.01
308.15	− 1.147 ± 0.105	112.029 ± 0.010	1.575	0.01
313.15	− 2.032 ± 0.149	112.584 ± 0.014	1.646	0.01
318.15	− 3.478 ± 0.101	113.122 ± 0.009	1.773	0.01
*d-*Fructose in aqueous solutions of [Ch][Ace] (0.0592 mol.kg^−1^ )				
298.15	− 0.716 ± 0.088	110.958 ± 0.008	1.502	0.01
303.15	− 1.319 ± 0.359	111.592 ± 0.035	1.645	0.03
308.15	− 1.176 ± 0.391	112.081 ± 0.038	1.627	0.03
313.15	− 2.479 ± 0.422	112.653 ± 0.041	1.715	0.04
318.15	− 3.866 ± 0.334	113.276 ± 0.032	1.927	0.03
*d-*Fructose in aqueous solutions of [Ch][Ace] (0.0888 mol.kg^−1^)				
298.15	− 7.555 ± 0.147	112.131 ± 0.014	2.675	0.01
303.15	− 7.022 ± 0.115	112.554 ± 0.011	2.607	0.01
308.15	− 6.272 ± 0.272	113.062 ± 0.026	2.608	0.02
313.15	− 7.125 ± 0.081	113.554 ± 0.007	2.616	0.01
318.15	− 6.073 ± 0.440	113.882 ± 0.043	2.533	0.04

^a^The standard uncertainties for molality, temperature and pressure were* u* (*m*) = 0.001 mol kg^−1^, *u* (*T*) = 0.2 K, *u* (*P*) = 10.5 hPa, respectively with level of confidence 0.95

Here, the least-square fitting of standard partial molar volumes at the temperatures under investigation yields the empirical constants *A, B,* and *C*. Standard apparent molar expansibilities ($E_{\varphi }^{0}$) was calculated using Eq. 3’s temperature derivative at constant pressure ((∂$V_{\varphi }^{0}$/∂T)_P_). In Table S2, the results are displayed for *d-*fructose in aqueous IL solutions which the computed values show a positive values. This positive standard apparent molar expansibilities is a feature of hydrophobic hydration solutions. Positive valuesare due to the volume of the solution grows more quickly than that of pure water. In the literature, this phenomenon has been thoroughly examined. As the temperature and IL concentration rise, the values show a positive trend and an increasing trend. This implies that the systems are dependent on temperature, particularly higher temperatures lead to greater molecular mobility. Frequently referred to as Hepler’s constant, the second derivative of $V_{\varphi }^{0}$ with respect to temperature represents the structure breaking or making behavior of *d-*fructose in the presence of aqueous ionic liquid solutions [13]:

4 $$\left( {\partial E_{\varphi }^{0} /\partial T} \right)_{P} = \left( {\partial^{2} V_{\varphi }^{0} /\partial T^{2} } \right)_{P} = 2C$$

Table S2 lists the calculated Hepler’s constants for the studied systems. Negative values of these constant imply that *d-*fructose acts as a structure breaker in aqueous IL environments, whereas positive values of the Hepler’s constants are an indicator of the structure-making behavior of the *d-*fructose in the studied solutions [40].

Notably, *d-*fructose’s Hepler’s constants in pure water are negative and almost zero, indicating that the sugar primarily plays a structural role in this setting. The following formula was used to determine the transfer volumes ($\Delta_{tr} V_{\varphi }^{0}$) of *d-*fructose from water to aqueous IL solutions at infinite dilution in order to exclude the impact of solute–solute and solvent–solvent interactions [13]:

5 $$\Delta_{tr} V_{\varphi }^{0} = V_{\varphi }^{0} \left( {Ternary} \right) - V_{\varphi }^{0} \left( {Binary} \right)$$

At infinite dilution, the calculated $\Delta_{tr} V_{\varphi }^{0}$ values are tabulated in Table 3. The obtained $\Delta_{tr} V_{\varphi }^{0}$ values consistently increase with rising IL molality. According to the co-sphere overlap model for ternary mixtures, interactions between IL species and water can be categorized into four types: a) hydrophilic–ionic, b) hydrophilic–hydrophilic, c) hydrophilic–hydrophobic, and d) hydrophobic–hydrophobic [14].

According to this model, interactions (c) and (d) gives negative $\Delta_{tr} V_{\varphi }^{0}$ values, but interactions (a) and (b) contribute to positive $\Delta_{tr} V_{\varphi }^{0}$ values. The obtained positive $\Delta_{tr} V_{\varphi }^{0}$ values imply that the majority of interactions between co-sphere molecules and polar groups or IL ions are hydrophilic. Moreover, heightened interactions of this kind are indicated by the growing tendency at higher IL concentrations. As a result, there is a clear complicated interaction between the co-solvent (ionic liquids) and solute (*d-*fructose) species.

### Acoustic results

The following expression was utilized to estimate the apparent molar isentropic compression ($\kappa_{\varphi }$) for *d-*fructose in aqueous IL solutions at different temperatures [15]:

6 $$\kappa_{\varphi } = (\frac{{M\kappa_{s} }}{\rho }) - \left[ {\frac{{\kappa_{s.0} \rho - \kappa_{s} \rho_{0} }}{{m\rho \rho_{0} }}} \right]$$

here *m* is *d-*fructose molality in the aqueous IL solution, *M* is the *d-*fructose molar mass, $\rho$ represents the density of the *d-*fructose in aqueous IL solutions, $\rho_{0}$ and represents the density of _*D-*_fructose in water, respectively. Using the following formula, the isentropic compressibility of the solutions and pure solvent were determined.

The density of *d-*fructose in aqueous ILs solutions has been indicated by $\rho$, the density of _*D-*_fructose in water by, $\rho_{0}$ and the molality of *d-*fructose in the aqueous IL solution by *m*, *M* is its molar mass. The following formula was used to determine the solutions’ and the pure solvent’s isentropic compressibility [15]:

7 $$\kappa_{s} = \frac{1}{{u^{2} \rho }}$$

**Fig. 3:**
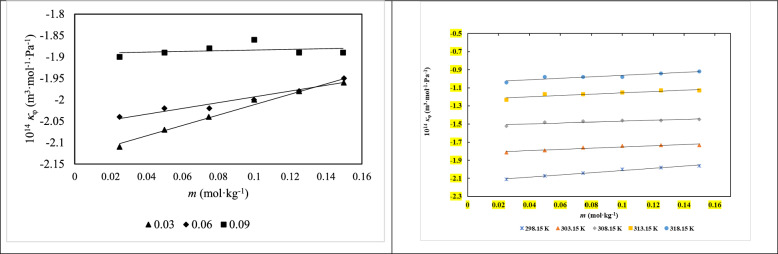
Apparent molar isentropic compressibility of *d-*fructose versus its molality *m* (mol.kg^−1^) in aqueous [Ch][Ace] solutions: (**a**) Different [Ch][Ace] molalities at *T* = 298.15 K and (**b**) A fixed [Ch][Ace] molality of 0.0300 mol kg^−1^ at different temperatures

The apparent molar isentropic compressibility $\kappa_{\varphi }$ values were obtained by utilizing the above expression, incorporating speed of sound (*u*) and density (*ρ*) of the *d-*fructose in the studied solutions. Table S3 displays the final *d-*fructose $\kappa_{\varphi }$ values in aqueous ILs solutions (at concentrations of 0.03, 0.06, and 0.09 mol∙kg⁻^1^) for each of the experimental temperatures. In general, as temperature and the amount of *d-*fructose and ionic liquids increased, so did the speed of sound. The graphical representation of the $\kappa_{\varphi }$ for *d-*fructose in aqueous ILs solution at various aqueous [Ch][Ace] solution concentrations has been depicted in Fig. 3. According to the results, $\kappa_{\varphi }$ values show a decreasing trend at all temperatures under study and get more negetive as the concentration of ILs rises. Aqueous solutions generally show (a) high negative values for ionic substances, (b) positive values for predominantly hydrophobic solutes, and (c) intermediate, tiny, and negative values for uncharged hydrophilic solutes such sugars, according to the literature [43, 44].

The variation of $\kappa_{\varphi }$ with respect to molality *m* an be effectively described using the following mathematical expression [15]:

8 $$\kappa_{\varphi } = \kappa_{\varphi }^{0} + S_{k} m$$

While $\kappa_{\varphi }^{0}$ is the standard partial molar isentropic compressibility value, $S_{k}$ has empirical interpretations that are comparable to those found in Eq. 2 for apparent molar volumes. Table 4 tabulates the values of $\kappa_{\varphi }^{0}$, $S_{k}$, and their standard deviations for the investigated solutions at the experimental temperatures. Due to the strong attractive interactions between *d-*fructose and ionic liquid, *d-*fructose $\kappa_{\varphi }^{0}$ values have been reported to increase as temperature and ionic liquid concentration increases. Using the following formula, the transfer partial molar isentropic compressibility ($\Delta_{tr} \kappa_{\varphi }^{0}$) of *d-*fructose from water to aqueous ILs solutions at infinite dilution was determined [15]:

9 $$\Delta_{tr} \kappa_{\varphi }^{0} = \kappa_{\varphi }^{0} \left( {{\text{Ternary }} {\text{}} {\text{}}} \right) - \kappa_{\varphi }^{0} \left( {{\text{Binary}} {\text{}}} \right)$$

**Table 4:** The values of partial molar isentropic compressibility ($\kappa_{\varphi }^{0}$), experimental slope ($S_{k}$), partial molar isentropic compressibility of transfer ($\Delta_{tr} \kappa_{\varphi }^{0}$) for *d-*fructose in the aqueous solutions of ILs at different temperature

*T* (K)	10^14^ *S*_k_ (m^3^·kg·mol^−2^·Pa^−1^)	10^14^ *κ*_φ_^0^ (m^3^·mol^−1^·Pa^−1^)	Δ*κ*_φ_^0^ (m^3^·mol^−1^·Pa^−1^)	*σ* (*κ*_φ_)
*d-*Fructose in water				
298.15	2.19 ± 0.206	− 2.35 ± 0.020	–	0.02
303.15	1.85 ± 0.173	− 2.04 ± 0.017	–	0.02
308.15	1.63 ± 0.175	− 1.78 ± 0.017	–	0.02
313.15	1.62 ± 0.165	− 1.51 ± 0.016	–	0.01
318.15	1.41 ± 0.281	− 1.26 ± 0.027	–	0.02
*d-*Fructose in aqueous solutions of [Ch][Sal]				
*m*_2_^*^ = (0.0308) mol·kg^−1^				
298.15	2.95 ± 0.454	− 2.37 ± 0.044	− 0.02	0.04
303.15	3.21 ± 0.267	− 2.11 ± 0.026	− 0.07	0.02
308.15	2.75 ± 0.193	− 1.80 ± 0.019	− 0.02	0.02
313.15	2.86 ± 0.253	− 1.54 ± 0.024	− 0.03	0.02
318.15	2.89 ± 0.442	− 1.29 ± 0.043	− 0.03	0.04
*d-*Fructose in aqueous solutions of [Ch][Sal]				
*m*_2_^*^ = (0.0603) mol·kg^−1^				
298.15	0.17 ± 0.166	− 1.94 ± 0.016	0.41	0.01
303.15	0.81 ± 0.219	− 1.80 ± 0.021	0.24	0.02
308.15	1.32 ± 0.190	− 1.62 ± 0.019	0.16	0.02
313.15	1.46 ± 0.322	− 1.43 ± 0.031	0.08	0.03
318.15	1.52 ± 0.072	− 1.24 ± 0.007	0.02	0.01
*d-*Fructose in aqueous solutions of [Ch][Sal]				
*m*_2_^*^ = (0.0899) mol·kg^−1^				
298.15	1.19 ± 0.381	− 2.11 ± 0.037	0.24	0.03
303.15	2.19 ± 0.265	− 1.97 ± 0.026	0.07	0.02
308.15	2.88 ± 0.176	− 1.83 ± 0.017	− 0.05	0.02
313.15	3.12 ± 0.201	− 1.64 ± 0.020	− 0.13	0.02
318.15	3.39 ± 0.211	− 1.47 ± 0.020	− 0.21	0.02
*d-*Fructose in aqueous solutions of [Ch][For]				
*m*_2_^*^ = (0.0287) mol·kg^−1^				
298.15	0.131 ± 0.350	− 2.101 ± 0.034	0.15	0.030
303.15	0.157 ± 291	− 1.790 ± 0.028	0.25	0.025
308.15	0.150 ± 0.487	− 1.512 ± 0.047	0.27	0.041
313.15	0.403 ± 250	− 1.324 ± 0.024	0.19	0.021
318.15	0.658 ± 0.383	− 1.160 ± 0.037	0.10	0.033
*d-*Fructose in aqueous solutions of [Ch][For]				
*m*_2_^*^ = (0.0593) mol·kg^−1^				
298.15	0.843 ± 0.362	− 2.150 ± 0.035	0.10	0.031
303.15	0.815 ± 0.281	− 1.852 ± 0.027	0.19	0.024
308.15	0.637 ± 0.160	− 1.549 ± 0.016	0.23	0.014
313.15	0.840 ± 0.279	− 1.315 ± 0.027	0.19	0.024
318.15	0.944 ± 0.299	− 1.099 ± 0.029	0.16	0.026
*d-*Fructose in aqueous solutions of [Ch][For]				
*m*_2_^*^ = (0.0889) mol·kg^−1^				
298.15	1.096 ± 0.201	− 2.095 ± 0.020	0.16	0.017
303.15	1.343 ± 0.141	− 1.911 ± 0.014	0.13	0.012
308.15	1.261 ± 0.255	− 1.722 ± 0.022	0.06	0.019
313.15	1.102 ± 0.213	− 1.510 ± 0.021	0.00	0.018
318.15	1.115 ± 0.234	− 1.331 ± 0.023	− 0.07	0.02
*d-*Fructose in aqueous solutions of [Ch][Ace]				
*m*_2_^*^ = (0.0292) mol·kg^−1^				
298.15	1.232 ± 0.072	− 2.137 ± 0.007	0.11	0.006
303.15	0.707 ± 0.082	− 1.822 ± 0.008	0.22	0.007
308.15	0.512 ± 0.120	− 1.518 ± 0.012	0.26	0.01
313.15	0.729 ± 0.161	− 1.227 ± 0.016	0.28	0.014
318.15	0.821 ± 0.154	− 1.044 ± 0.015	0.22	0.013
*d-*Fructose in aqueous solutions of [Ch][Ace]				
*m*_2_^*^ = (0.0595) mol·kg^−1^				
298.15	0.672 ± 0.085	− 2.061 ± 0.008	0.19	0.007
303.15	0.794 ± 0.217	− 1.765 ± 0.021	0.28	0.019
308.15	1.271 ± 0.270	− 1.538 ± 0.026	0.24	0.023
313.15	0.363 ± 0.067	− 1.176 ± 0.060	0.33	0.005
318.15	0.693 ± 0.384	− 1.003 ± 0.037	0.26	0.033
*d-*Fructose in aqueous solutions of [Ch][Ace]				
*m*_2_^*^ = (0.0892) mol·kg^−1^				
298.15	0.075 ± 0.144	− 1.893 ± 0.014	0.36	0.012
303.15	0.580 ± 0.172	− 1.632 ± 0.017	0.41	0.015
308.15	0.360 ± 0.233	− 1.345 ± 0.023	0.43	0.020
313.15	0.231 ± 0.039	− 1.068 ± 0.003	0.44	0.003
318.15	0.449 ± 0.025	− 0.894 ± 0020	0.37	0.002

m_2_^*^: molality aqueous ILs solution

^a^The standard uncertainties for molality, temperature and pressure were* u* (*m*) = 0.001 mol kg^−1^, *u* (*T*) = 0.2 K, *u* (*P*) = 10.5 hPa, respectively with level of confidence 0.95

The computed $\Delta_{tr} \kappa_{\varphi }^{0}$ results are provided in Table 4. The presence of positive $\Delta_{tr} \kappa_{\varphi }^{0}$ values suggests that type (a) and type (b) interactions, based on the co-sphere overlap model, are dominant. The increasingly negative values of apparent molar compressibility with rising IL concentration suggest that pressure enhances a repulsive effect in the bulk phase, attributed to the solvation of *d-*fructose. Nonetheless, the incorporation of ionic liquids enhances the overall compressibility of the system. Overall, the findings point to weak electrostatic interactions between the ILs and *d-*fructose. Changes in the bulk modulus, partial dehydration of ionic species, expansion of solution volume, and a decrease in the hydration shell surrounding both *d-*fructose and the IL ions all contribute to the reinforcement of these interactions at high temperatures.

### Viscosity *B*-coefficients

**Fig. 4:**
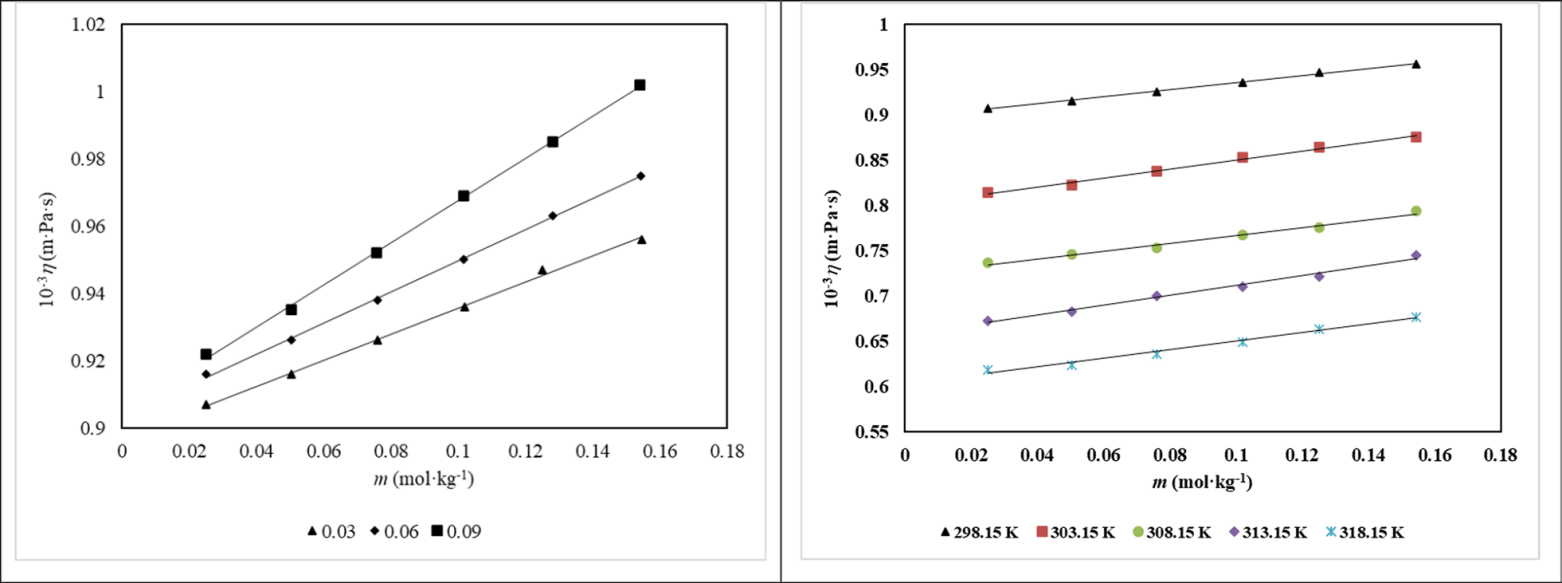
The viscosity of aqueous *d-*fructose solutions versus their molality, *m*, (mol.kg^−1^) in aqueous [Ch][Ace] solutions **a** Different [Ch][Ace] molalities at *T* = 298.15 K and **b** A fixed [Ch][Ace] molality of 0.0300 at varied temperatures

Table S4 and Fig. 4 illustrate the experimental viscosity data (*η*) for *d-*fructose in pure water and in aqueous solutions containing three choline-based ILs at concentrations of 0.03, 0.06, and 0.09 mol∙kg⁻^1^ across the temperature range from (298 to 318) K. The viscosity increases with the molar mass of the ILs but decreases as temperature rises, consistent with typical thermal behavior. Moreover, viscosity shows a positive correlation with increasing concentrations of both *d-*fructose and ionic liquids. The observed changes in relative viscosity (*η*_r_) for *d-*fructose in water and IL-containing aqueous media were interpreted using the Jones–Dole equation [13]:

10 $$\frac{\eta }{{\eta_{0} }} = 1 + Ac^{1/2} + Bc$$

Two crucial factors in the Jones-Dole equation are employed to characterize solute–solvent interactions: the viscosity *B*-coefficient (*B*) and the Falkenhagen coefficient (*A*). Among these, the *B*-coefficient plays a significant role, as it reflects the influence of solute properties such as size, shape, and charge on the solution’s viscosity. In contrast, the Falkenhagen coefficient, which accounts for solute–solute interactions, was determined to be negligible in the studied systems based on least-squares fitting. This negligible contribution indicates weak solute–solute interactions, allowing the equation to be simplified by omitting the Falkenhagen term, thus yielding the standard form of the viscosity equation [13]:

11 $$\frac{\eta }{{\eta_{0} }} = 1 + Bc$$

here, *η* and *η*_₀_ stand for the viscosities of the solvent (aqueous ILs solutions) and the ternary systems containing *d-*fructose in aqueous ILs, respectively, and *c* for the molar concentration of *d-*fructose in the aqueous ILs solutions. The viscosity *B*-coefficients were determined from the slope of the linear plot of (*η*_r_ − 1) versus *c*, using the least-squares fitting method. The resulting *B*-coefficients and *η*_r_ values, derived from fitting the experimental viscosity data to the simplified Jones–Dole equation, are presented in Table 5. The viscosity *B*-coefficient offers valuable insights into solute characteristics—including size, shape, and charge—as well as the structural modifications induced by solute–solvent interactions [16, 17]. A measure of solvation and its impact on the surrounding solvent structure is the viscosity *B*-coefficient. It shows how hydrophilic or hydrophobic moieties and charged end groups collectively affect the way solvent molecules are arranged. The significant kosmotropic (structure-making) action in aqueous IL solutions is indicated by the positive *B*-coefficient values for *d-*fructose. Strong solute–solvent interactions are implied by this, underscoring *d-*fructose’s capacity to improve solvent network structure in the presence of ionic liquids. Numerous studies have reported that the *dB/dT* value is a more effective criterion for determining the structure-making or structure-breaking nature of a solute than the viscosity *B*-coefficient. According to Table 5, the values of *dB/dT* in most cases are positive, indicating that *D*-fructose has a tendency to break the structure in the systems studied. These findings are consistent with the results obtained from Hepler's constant and complement each other well.

**Table 5:** The viscosity *B*-coefficient, dB/dT, and the standard deviation of the viscosity *σ*(*η*) data value for *d-*fructose in water and *d-*fructose in aqueous ILs solutions at (298–318) K and 871 hPa

*T* (K)	*B* (dm^3/2^·mol^−1/2^)	*10*^3^*(dB/dT)* (dm^3/2^·mol^−1/2^ K^-1^)	*σ* (*η*)
*d-*Fructose in aqueous solutions of [Ch][Sal] (0.0298 mol·kg^−1^)			
298.15	0.415 ± 0.053	0.204 ± 0.023	0.02
303.15	0.417 ± 0.041	0.938 ± 0.033	0.01
308.15	0.425 ± 0.033	1.704 ± 0.052	0.01
313.15	0.434 ± 0.060	2.503 ± 0.069	0.05
318.15	0.449 ± 0.047	3.334 ± 0.074	0.07
*d-*Fructose in aqueous solutions of [Ch][Sal] (0.0597 mol·kg^−1^)			
298.15	0.542 ± 0.095	0.480 ± 0.041	0.09
303.15	0.508 ± 0.064	0.370 ± 0.001	0.07
308.15	0.856 ± 0.028	0.252 ± 0.036	0.05
313.15	1.051 ± 0.075	0.130 ± 0.034	0.02
318.15	0.893 ± 0.021	0.331 ± 0.064	0.03
*d-*Fructose in aqueous solutions of [Ch][Sal] (0.0899 mol·kg^−1^)			
298.15	0.494 ± 0.065	− 5.042 ± 0.082	0.50
303.15	0.468 ± 0.042	− 6.448 ± 0.079	0.10
308.15	0.434 ± 0.085	− 7.917 ± 0.028	0.04
313.15	0.382 ± 0.061	− 9.450 ± 0.034	0.06
318.15	0.338 ± 0.006	− 11.046 ± 0.016	0.01
*d-*Fructose in aqueous solutions of [Ch][For] (0.0299 mol·kg^−1^)			
298.15	0.475 ± 0.052	6.865 ± 0.018	0.01
303.15	0.809 ± 0.047	4.779 ± 0.024	0.04
308.15	1.030 ± 0.013	2.579 ± 0.062	0.09
313.15	0.947 ± 0.011	0.265 ± 0.093	0.05
318.15	1.035 ± 0.066	− 2.163 ± 0.070	0.02
*d-*Fructose in aqueous solutions of [Ch][For] (0.0596 mol·kg^−1^)			
298.15	0.563 ± 0.090	3.654 ± 0.001	0.07
303.15	0.775 ± 0.036	3.172 ± 0.036	0.05
308.15	0.963 ± 0.004	2.661 ± 0.102	0.01
313.15	0.918 ± 0.025	2.119 ± 0.039	0.06
318.15	1.152 ± 0.069	1.548 ± 0.068	0.03
*d-*Fructose in aqueous solutions of [Ch][For] (0.0899 mol·kg^−1^)			
298.15	0.753 ± 0.063	0.879 ± 0.070	0.02
303.15	0.861 ± 0.096	2.225 ± 0.033	0.03
308.15	0.965 ± 0.030	3.635 ± 0.028	0.01
313.15	1.207 ± 0.028	5.109 ± 0.045	0.02
318.15	1.498 ± 0.068	6.649 ± 0.061	0.04
*d-*Fructose in aqueous solutions of [Ch][Ace] (0.0298 mol·kg^−1^)			
298.15	0.475 ± 0.050	3.294 ± 0.090	0.03
303.15	0.667 ± 0.043	3.231 ± 0.060	0.01
308.15	0.813 ± 0.006	3.160 ± 0.150	0.06
313.15	0.940 ± 0.014	3.080 ± 0.054	0.03
318.15	1.128 ± 0.063	2.991 ± 0.125	0.05
*d-*Fructose in aqueous solutions of [Ch][Ace] (0.0596 mol·kg^−1^)			
298.15	0.563 ± 0.080	3.654 ± 0.008	0.01
303.15	0.775 ± 0.061	3.172 ± 0.026	0.01
308.15	0.963 ± 0.015	2.661 ± 0.037	0.02
313.15	0.918 ± 0.042	2.119 ± 0.106	0.07
318.15	1.152 ± 0.072	1.548 ± 0.111	0.03
*d-*Fructose in aqueous solutions of [Ch][Ace] (0.0897 mol·kg^−1^)			
298.15	0.753 ± 0.036	0.879 ± 0.004	0.02
303.15	0.861 ± 0.043	2.225 ± 0.082	0.05
308.15	0.965 ± 0.074	3.635 ± 0.251	0.01
313.15	1.207 ± 0.060	5.109 ± 0.163	0.03
318.15	1.498 ± 0.072	6.649 ± 0.121	0.07

^a^The standard uncertainties for molality, temperature and pressure were* u* (*m*) = 0.001 mol kg^−1^, *u* (*T*) = 0.2 K, *u* (*P*) = 10.5 hPa, respectively with level of confidence 0.95

### Electrical conductivity results

**Fig. 5:**
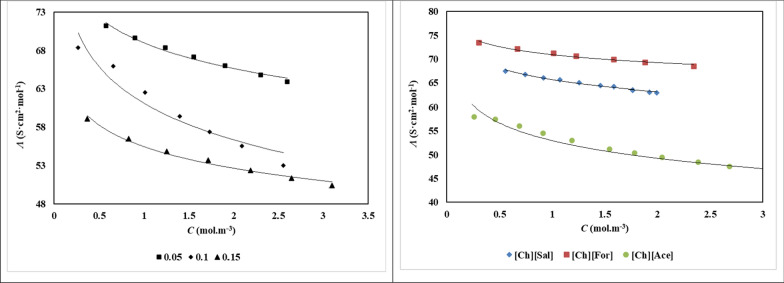
The molar conductivities (*Λ*) of [Ch][Ace] in aqueous *d-*fructose solutions with different molality concentrations of *d-*fructose: ■, 0.0500; ◆, 0.1000; ▲, 0.1500 at *T* = 298 K

The molar conductivity ($\Lambda$) values of choline based ILs in aqueous solutions of *d-*fructose have been given in Table S5. Figure 5 graphically illustrates the dependence of molar conductivity (Λ) of the ionic liquid (IL) at various *d-*fructose molalities. A clear decreasing trend is observed, where molar conductivity diminishes with increasing concentrations of both *d-*fructose and ILs. This behavior can be attributed to enhanced ion–solute and ion–solvent interactions, which hinder the mobility of charge carriers. To further interpret the experimental data, the low concentration Chemical Model (lcCM) was employed, utilizing the following equations [19]:

12 $$\Lambda = \alpha \left[ {\Lambda_{0} - S\left( {c\alpha } \right)^{{{\raise0.7ex\hbox{$1$} \!\mathord{\left/ {\vphantom {1 2}}\right.\kern-0pt} \!\lower0.7ex\hbox{$2$}}}} + Ec\alpha \ln (c\alpha ) + J_{1} c\alpha + J_{2} (c\alpha )^{{{\raise0.7ex\hbox{$3$} \!\mathord{\left/ {\vphantom {3 2}}\right.\kern-0pt} \!\lower0.7ex\hbox{$2$}}}} } \right]$$

13 $$K_{A} = \frac{1 - \alpha }{{\alpha^{2} c\gamma_{ \pm }^{2} }}$$

14 $$\ln \gamma_{ \pm } = - \frac{kq}{{1 + kR}}$$

15 $$k^{2} = \frac{{16000N_{A} z^{2} e^{2} \alpha c}}{{\varepsilon_{0} \varepsilon k_{B} T}}$$

16 $$q = \frac{{z^{2} e^{2} }}{{8\pi \varepsilon_{0} \varepsilon k_{B} T}}$$

where *Λ*, $\Lambda_{0}$ are the molar conductivity and limiting molar conductivity values, ($1 - \alpha$) is the fraction of oppositely charged ions existing as free ions (i.e., not associated as ion pairs), distance parameter (*R*) associated with the effective separation of ion pairs, and mean activity coefficient (*γ*_±_) of the free ions. Based on the information provided in the literature, the other parameters required to calculate *α*, *Λ⁰*, and *R* were acquired [19].

*Here, C* represents the molar concentration of *d-*fructose in this equation, which has been determined by the density and molality values of the solution. The meanings of the other parameters stay the same. Table 6 tabulates the determined *K*_A_, *Λ*_0_, and *R* which has been obtained by applying a nonlinear least-squares iteration to the molar conductivity data. Both (i) stronger interactions between *d-*fructose and IL ions, which result in larger solvated ion radii and decreased mobility, and (ii) increased solution viscosity due to higher IL content, which hinders ion mobility, are the main causes of the observed decrease in *K*_A_ and increase in *K*_A_ with rising IL concentration. *C* represents the molar concentration of *d-*fructose in this equation, which is determined by the molality and related density values of the solution.

**Table 6:** The association constants (*K*_*A*_), limiting molar conductivities ($\Lambda_{0}$), the distance parameter (*R*), and standard deviations (*S*_dev_(*Λ*)) of choline based ionic liquids in aqueous *d-*fructose solutions at 298 K

*m* (mol·kg^−1^)	*K*_A_ (dm^3^·mol^−1^)	*Λ*_0_ (S.cm^2^.mol^−1^)	10^10^*R* (m)	*S*_dev_(Λ)
[Ch][Sal] in aqueous *d-*fructose				
0.0000	41.300 ± 1.713	70.135 ± 0.150	34.11	0.11
0.0488	49.309 ± 1.899	69.307 ± 0.160	35.32	0.15
0.0998	55.852 ± 2.674	67.208 ± 0.207	35.86	0.14
0.1495	58.008 ± 1.428	64.759 ± 0.111	30.58	0.05
[Ch][For] in aqueous *d-*fructose				
0.0000	37.073 ± 2.620	74.616 ± 0.225	26.47	0.07
0.0509	59.373 ± 3.391	74.489 ± 0.295	33.51	0.06
0.1004	61.409 ± 2.798	71.82 ± 0.213	50.44	0.48
0.1496	67.166 ± 3.351	60.75 ± 0.247	34.14	0.32
[Ch][Ace] in aqueous *d-*fructose				
0.0000	124.099 ± 2.061	59.562 ± 0.125	6.52	0.26
0.0499	114.228 ± 4.407	56.298 ± 0.302	6.67	0.20
0.0998	72.037 ± 2.391	50.909 ± 0.188	27.79	0.12
0.1498	49.705 ± 2.598	48.999 ± 0.210	30.95	0.08

The estimated uncertainities for u (*K*_*A*_) = 0.3 dm^3^·mol, *u* (10^4^*Λ*_*0*_) = 0.075 S·m^2^·mol. (level of confidence 0.68)

The definitions of the other parameters are still standard. Table 6 summarizes the findings of the nonlinear least-squares fitting of the experimental molar conductivity data to estimate the ion-association constant (*K*ₐ), limiting molar conductivity (*Λ*_⁰_), and distance parameter (*R*). Two primary factors account for the observed decrease in *Λ* and the corresponding rise in *K*ₐ as IL concentration rises: (i) increased interactions between *d-*fructose and IL ions, which increase the effective solvation shell around ions and decrease their mobility; and (ii) increased solution viscosity at higher IL concentrations, which further hinders ion transport [20, 21]. A contributing factor to ion association is the increased electrostatic contacts between ILs and *d-*fructose that result from the higher IL ion count. Additionally, the higher solution viscosity decreases ionic diffusion and mobility, which encourages the formation of ion pairs in the systems under study.

### Taste behavior

The volumetric behavior and taste perception of monosaccharides in complex solvent systems have been explored in recent studies, the main research included those that contained deep eutectic solvents (DESs) and ionic liquids (ILs) [22, 23]. In such systems, apparent specific volume (*ASV*) and apparent specific isentropic compressibility (*ASIC*) are widely used to infer molecular-level interactions and hydration characteristics. These parameters provide valuable insight into how solutes like sugars integrate into the water structure and how such integration affects perceived sweetness, which is closely linked to the solute’s hydration shell and structural compatibility with the solvent medium.

Research on the hydration properties of taste molecules, particularly sweeteners, has revealed important insights into taste perception. The apparent specific volume (*ASV*) of sweeteners, typically ranging from 0.55 to 0.68 cm^3^·g⁻^1^, is a key determinant of taste quality. Aroulmoji et al. [24]. reported that the *ASV* of sucrose in aqueous solutions (approximately 0.626 cm^3^·g⁻^1^) falls within the empirical “sweet taste range,” indicating its high structural compatibility with water and efficient hydration [24, 25]. *ASV* values within or above this range suggest that the solute has a greater influence on structuring the solvent environment, thereby enhancing its sweetness perception. Conversely, lower *ASV* values could imply less favorable interactions with the aqueous medium and reduced taste intensity [24, 25].

In the present study, we extend this framework to *d-*fructose, a monosaccharide known for its high intrinsic sweetness. The aim is to understand how choline-based ILs, with varying alkyl chain lengths, influence the volumetric and taste-related properties of d-fructose in aqueous media. To this end, *ASV* and *ASIC* values were computed using the Eqs. 17 and 18 respectively. The *ASV* is primarily influenced by solute–solvent interactions and can reflect changes in the hydration shell structure around *d-*fructose molecules. An increase in *ASV* with IL concentration may indicate enhanced solute–solvent compatibility due to the kosmotropic (structure-making) nature of the ILs, which promotes stronger hydrogen bonding and organized hydration layers around *d-*fructose [27, 28].

Similarly, the *ASIC* serves as a complementary parameter to evaluate the compressibility of the solute–solvent system, which can be linked to solution cohesiveness and structural integrity. Lower ASIC values suggest a more rigid and structured hydration shell, typically associated with stronger solute–solvent interactions and a potential enhancement in sweetness perception [30–32].

The resulting *ASV* and *ASIC* values for *d-*fructose in both pure water and aqueous IL solutions at varying temperatures and concentrations are summarized in Table S6. These data collectively provide a thermodynamic and structural basis to understand how ILs modulate the hydration environment and possibly the sensory perception of *d-*fructose [34].

17 $$ASV = \frac{{V_{\varphi } }}{M}$$

18 $$ASIC = \frac{{\kappa_{\varphi } }}{M}$$

*d-*Fructose’s *ASV* and *ASIC* values in aqueous IL solutions and pure water (Table S6) indicate that the addition of the investigated ILs does not substantially change the *d-*fructose’s physical characteristics associated with its flavor [34].

### Hydration number results

By utilizing Eq. 18, the hydration numbers (*n*_H_) of *d-*fructose in aqueous ionic liquid (IL) solutions and pure water are reported in Table S7. Hydration number, as a representative of the number of water molecules in the immediate hydration shell of *d-*fructose, is strongly related to the volume change caused by electrostriction, which represents the contraction of water molecules around solute species [27, 35, 36]. Because hydration is dynamic and non-stoichiometric, measuring hydration numbers accurately is still a challenging endeavor, despite the fact that several structural and computational methods have been used to investigate solute–solvent interactions. Using the following connection, hydration numbers were obtained from thermodynamic data in the current work [37]:

19 $$n_{H} = \frac{{V_{\phi }^{0} \left( {elect.} \right)}}{{V_{E}^{0} - V_{B}^{0} }}$$

where $V_{\phi }^{0} \left( {elect.} \right)$ indicates the electrostriction partial molar volume produced by *d-*fructose hydration. $V_{\phi }^{0} \left( {elect.} \right)$ can be estimated using *d-*fructose’s intrinsic partial molar volume ($V_{\phi }^{0}$(int.)) and its corresponding value, as per the following formula [4]:

20 $$V_{\phi }^{0} \left( {elect.} \right) = V_{\phi }^{0} - V_{\phi }^{0} ({\text{int}} .)$$

where,

21 $$V_{\phi }^{0} ({\text{int}} .) = \left( {\frac{0.7}{{0.634}}} \right).V_{\phi }^{0} (cryst.)$$

22 $$V_{\phi }^{0} (cryst.) = \left( {{\raise0.7ex\hbox{$M$} \!\mathord{\left/ {\vphantom {M {d_{cryst.} }}}\right.\kern-0pt} \!\lower0.7ex\hbox{${d_{cryst.} }$}}} \right)$$

In the calculation, a packing density of 0.7 was employed for molecules in organic crystals, while a value of 0.634 was used for randomly packed spheres. The crystal molar volume $V_{\phi }^{0} (cryst.)$ of *d-*fructose was computed using its molar mass (*M*) and a crystalline density ($d_{cryst.}$) of 1.544 g·cm⁻^3^. Among the key parameters influencing the estimation of the hydration number ($V_{E}^{0} - V_{B}^{0}$) is the electrostriction partial molar volume, which accounts for the volumetric contraction arising from solute–solvent interactions. For *d-*fructose, this parameter exhibited temperature dependence, with values of − 3.3, − 3.61, − 4.00, − 4.35, and − 4.65 cm^3^·mol⁻^1^ at 298.15, 303.15, 308.15, 313.15, and 318.15 K, respectively [4, 25, 37].

The linear regression analysis was applied to estimate the electrostriction partial molar volume values at 303.15, 313.15, and 318.15 K. In this context, $V_{B}^{0}$ refers to both the bulk molar volume and the electrostricted molar volume of water ($V_{E}^{0}$). These factors were added to Eq. (18) to determine the hydration numbers (n_H_) for *d-*fructose at different temperatures. According to Table S7, the results show that the hydration number gradually decreases as the temperature rises, emphasizing the enhanced dehydration impact that the ionic liquids produce at higher temperatures.

## Conclusions

In this work the interactions with three ionic liquids ([Ch][Sal], [Ch][For], and [Ch][Ace]) in aqueous solutions of *D*-fructose were investigated using electrical conductivity, volumetric, compressibility, and viscosity measurements. Standard and transfer partial molar characteristics of *d-*fructose in aqueous IL solutions were determined by calculating its apparent molar volume ($V_{\varphi }$) and apparent molar isentropic compressibility ($\kappa_{\varphi }$), which were derived from density and speed of sound data. According to the results, the interactions between *d-*fructose and ILs became more stronger as the concentration of IL increased. According to the derived transfer characteristics $\Delta_{tr} V_{\varphi }^{0}$ and $\Delta_{tr} \kappa_{\varphi }^{0}$, *d-*fructose and IL ions primarily interact hydrophilically with ionic and hydrophilically with hydrophilic ions. According to viscosity measurements, the order of the viscosity *B*-coefficient rose as the concentration of IL increased: [Ch][Sal] > [Ch][For] > [Ch][Ace].

The limiting molar conductivity ($\Lambda_{0}$) of *d-*fructose decreased with increasing concentrations of ionic liquids (ILs), while the ion-association constant (*K*_A_) exhibited a corresponding increase. This behavior is primarily attributed to enhanced electrostatic interactions and elevated solution viscosity at higher IL concentrations, which promote ion-pair formation and hinder ionic mobility.

The obtained *ASV* and *ASIC* values in this study suggests that the ILs employed in the current system are compatible and may serve as effective and environmentally benign additives in biotechnological processes such as bioethanol production.

Moreover, the physicochemical properties of the choline-based ILs were found to be strongly dependent on the length of their alkyl chains. Increases in chain length were associated with shifts in HOMO–LUMO energy levels and dielectric solvation energies, indicating modifications in the electronic structure and a corresponding enhancement in solute–solvent interactions.

The temperatures increment in the studied systems leads to an increase in the kinetic energy of water molecules, leading to weakened hydrogen bonding with *d-*fructose and, consequently, a reduction in hydration. This competitive effect becomes more pronounced with increasing IL concentration, further decreasing the hydration number.

## Supplementary Information

Additional file 1.

## Data Availability

The authors confirm that the data supporting the findings of this study are available within the manuscript, figures, tables and supporting information files.
