# Supplementary material for: The role of choline-based ionic liquids in modulating the thermophysical properties of d-fructose solutions
Source: BMC Chem. 2025 Jun 5;19(1):160. doi: 10.1186/s13065-025-01491-5 (PMC12142968; doi:10.1186/s13065-025-01491-5)
Supplement: Supplementary file 1 — Additional file 1. [file 13065_2025_1491_MOESM1_ESM.docx]

**Supporting Materials**

**The role of choline-based ionic liquids in modulating the thermophysical properties of *D*-fructose solutions**

**Sara Dorosti, Hemayat Shekaari[[1]](#footnote-1), Mohammad Bagheri, Fariba Ghaffari, Masumeh Mokhtarpour**

*Department of Physical Chemistry, University of Tabriz, Tabriz, Iran*

**Table S1.** The density, $\rho$*,* and apparent molar volume,, values for D-Fructose in the aqueous ILs solutions at various temperature.*^a^*

| *m*  (mol·kg^-1^) | 10^-3^·*ρ* (g·cm^-3^) | | | | |  | 10^6^·*Vϕ* (m^3^·mol^-1^) | | | |  |
| --- | --- | --- | --- | --- | --- | --- | --- | --- | --- | --- | --- |
| *T* (K) | 298.15 | 303.15 | 308.15 | 313.15 | 318.15 |  | 298.15 | 303.15 | 308.15 | 313.15 | 318.15 |
| D-Fructose in water | | | | | | | | | | |  |
| 0.0000 | 0.997042 | 0.995646 | 0.994023 | 0.992208 | 0.990201 |  |  |  |  |  |  |
| 0.0250 | 0.998791 | 0.997384 | 0.995750 | 0.993925 | 0.991910 |  | 110.104 | 110.604 | 111.115 | 111.597 | 112.008 |
| 0.0500 | 1.000524 | 0.999107 | 0.997460 | 0.995625 | 0.993603 |  | 110.284 | 110.764 | 111.317 | 111.8 | 112.192 |
| 0.0750 | 1.002236 | 1.000807 | 0.999154 | 0.997305 | 0.995272 |  | 110.425 | 110.927 | 111.4 | 111.939 | 112.38 |
| 0.1000 | 1.003926 | 1.002496 | 1.000829 | 0.998971 | 0.996927 |  | 110.649 | 111.051 | 111.566 | 112.082 | 112.549 |
| 0.1250 | 1.005624 | 1.004174 | 1.002487 | 1.000622 | 0.998575 |  | 110.657 | 111.153 | 111.743 | 112.229 | 112.645 |
| 0.1500 | 1.007297 | 1.005839 | 1.004150 | 1.002265 | 1.000208 |  | 110.775 | 111.253 | 111.77 | 112.326 | 112.756 |
| D-Fructose in aqueous solution of [Ch][Sal] (0.03 mol.kg^-1^) | | | | | | | | | | |  |
| 0.0000 | 0.998178 | 0.996848 | 0.995586 | 0.994396 | 0.993261 |  | - | - | - | - |  |
| 0.0249 | 0.999927 | 0.998584 | 0.997309 | 0.996103 | 0.994955 |  | 109.87 | 110.448 | 111.025 | 111.725 | 112.302 |
| 0.0501 | 1.001680 | 1.000322 | 0.999031 | 0.997802 | 0.996643 |  | 109.923 | 110.54 | 111.176 | 112.015 | 112.551 |
| 0.0747 | 1.003366 | 1.001982 | 1.000683 | 0.999443 | 0.998272 |  | 110.203 | 110.987 | 111.541 | 112.271 | 112.811 |
| 0.0998 | 1.005059 | 1.003683 | 1.002350 | 1.001108 | 0.999925 |  | 110.502 | 111.022 | 111.796 | 112.377 | 112.917 |
| 0.1249 | 1.006746 | 1.005346 | 1.004013 | 1.002753 | 1.001531 |  | 110.705 | 111.327 | 111.957 | 112.579 | 113.34 |
| 0.1468 | 1.008202 | 1.006771 | 1.005416 | 1.004117 | 1.002904 |  | 110.82 | 111.573 | 112.271 | 113.08 | 113.674 |
|  |  |  |  |  |  |  |  |  |  |  |  |
| D-Fructose in aqueous solution of [Ch][Sal] (0.06 mol.kg-1) | | | | | | | | | | |  |
| 0.0000 | 0.999463 | 0.998106 | 0.996806 | 0.995578 | 0.99440 |  | - | - | - | - |  |
| 0.0253 | 1.001211 | 0.999849 | 0.998540 | 0.997303 | 0.996120 |  | 110.791 | 111.046 | 111.46 | 111.872 | 112.123 |
| 0.0501 | 1.002919 | 1.001545 | 1.000230 | 0.998992 | 0.997796 |  | 110.833 | 111.231 | 111.588 | 111.842 | 112.257 |
| 0.0751 | 1.004625 | 1.003243 | 1.001925 | 1.000678 | 0.999473 |  | 110.903 | 111.295 | 111.592 | 111.901 | 112.316 |
| 0.1003 | 1.006329 | 1.00494 | 1.00361 | 1.002347 | 1.001143 |  | 110.945 | 111.323 | 111.681 | 112.088 | 112.403 |
| 0.1247 | 1.007981 | 1.006584 | 1.005251 | 1.003982 | 1.002766 |  | 110.924 | 111.305 | 111.628 | 112.015 | 112.376 |
| 0.1498 | 1.009664 | 1.008266 | 1.006903 | 1.005632 | 1.004409 |  | 110.966 | 111.3 | 111.782 | 112.127 | 112.485 |
|  |  |  |  |  |  |  |  |  |  |  |  |
| D-Fructose in aqueous solution of [Ch][Sal] (0.09 mol.kg^-1^) | | | | | | | | | | |  |
| 0.0000 | 1.000715 | 0.999353 | 0.998035 | 0.996779 | 0.995330 |  | - | - | - | - |  |
| 0.025 | 1.002440 | 1.001070 | 0.999745 | 0.998479 | 0.997022 |  | 110.921 | 111.299 | 111.636 | 112.093 | 112.48 |
| 0.0502 | 1.004161 | 1.002786 | 1.001451 | 1.000180 | 0.998707 |  | 111.039 | 111.356 | 111.753 | 112.109 | 112.657 |
| 0.0751 | 1.005860 | 1.004473 | 1.003138 | 1.001855 | 1.000383 |  | 111.062 | 111.454 | 111.738 | 112.156 | 112.53 |
| 0.0998 | 1.007538 | 1.006136 | 1.004791 | 1.003504 | 1.002012 |  | 111.016 | 111.476 | 111.806 | 112.175 | 112.675 |
| 0.1248 | 1.009214 | 1.007810 | 1.006450 | 1.005148 | 1.003635 |  | 111.096 | 111.493 | 111.889 | 112.317 | 112.902 |
| 0.1496 | 1.010857 | 1.009448 | 1.008070 | 1.006752 | 1.005252 |  | 111.209 | 111.585 | 112.047 | 112.523 | 112.935 |
|  |  |  |  |  |  |  |  |  |  |  |  |
| D-Fructose in aqueous solution of [Ch][For] (0.03 mol.kg-1) | | | | | | | | | | | |
| 0.0000 | 0.998178 | 0.996848 | 0.995586 | 0.994396 | 0.993261 |  | - | - | - | - | - |
| 0.0249 | 0.999927 | 0.998584 | 0.997309 | 0.996104 | 0.994955 |  | 109.87 | 110.45 | 111.03 | 111.68 | 112.30 |
| 0.0501 | 1.001680 | 1.000322 | 0.999031 | 0.997807 | 0.996643 |  | 109.92 | 110.54 | 111.18 | 111.91 | 112.55 |
| 0.0747 | 1.003366 | 1.001982 | 1.000683 | 0.999449 | 0.998272 |  | 110.20 | 110.99 | 111.54 | 112.19 | 112.81 |
| 0.0998 | 1.005059 | 1.003683 | 1.002350 | 1.001108 | 0.999925 |  | 110.50 | 111.02 | 111.80 | 112.38 | 112.92 |
| 0.1249 | 1.006746 | 1.005346 | 1.004013 | 1.002753 | 1.001541 |  | 110.71 | 111.33 | 111.96 | 112.58 | 113.26 |
| 0.1468 | 1.008202 | 1.006771 | 1.005436 | 1.004147 | 1.002944 |  | 110.82 | 111.57 | 112.13 | 112.87 | 113.40 |
|  |  |  |  |  |  |  |  |  |  |  |  |
| D-Fructose in aqueous solution of [Ch][For] (0.06 mol.kg^-1^) | | | | | | | | | | | |
| 0.0000 | 0.999463 | 0.998106 | 0.996806 | 0.995578 | 0.994400 |  |  |  |  |  |  |
| 0.0253 | 1.001211 | 0.999845 | 0.998536 | 0.997299 | 0.996112 |  | 110.79 | 111.21 | 111.62 | 112.03 | 112.44 |
| 0.0501 | 1.002919 | 1.001540 | 1.000225 | 0.998975 | 0.997776 |  | 110.83 | 111.33 | 111.69 | 112.19 | 112.66 |
| 0.0751 | 1.004625 | 1.003238 | 1.001911 | 1.000638 | 0.999423 |  | 110.90 | 111.36 | 111.78 | 112.44 | 112.99 |
| 0.1003 | 1.006329 | 1.004930 | 1.003591 | 1.002297 | 1.001063 |  | 110.95 | 111.42 | 111.87 | 112.59 | 113.21 |
| 0.1247 | 1.007971 | 1.006560 | 1.005206 | 1.003892 | 1.002651 |  | 111.00 | 111.50 | 111.99 | 112.75 | 113.31 |
| 0.1498 | 1.009644 | 1.008221 | 1.006848 | 1.005512 | 1.004269 |  | 111.10 | 111.60 | 112.15 | 112.94 | 113.44 |
|  |  |  |  |  |  |  |  |  |  |  |  |
| D-Fructose in aqueous solution of [Ch][For] (0.09 mol.kg^-1^) | | | | | | | | | | | |
| 0.0000 | 1.000715 | 0.999353 | 0.998035 | 0.996779 | 0.995330 |  |  |  |  |  |  |
| 0.0250 | 1.002440 | 1.001070 | 0.999743 | 0.998477 | 0.997017 |  | 110.92 | 111.30 | 111.72 | 112.17 | 112.68 |
| 0.0502 | 1.004165 | 1.002786 | 1.001447 | 1.000167 | 0.998697 |  | 110.96 | 111.36 | 111.83 | 112.37 | 112.86 |
| 0.0751 | 1.005860 | 1.004473 | 1.003120 | 1.001825 | 1.000343 |  | 111.06 | 111.45 | 111.98 | 112.56 | 113.07 |
| 0.0998 | 1.007528 | 1.006126 | 1.004761 | 1.003454 | 1.001962 |  | 111.12 | 111.58 | 112.11 | 112.68 | 113.18 |
| 0.1248 | 1.009204 | 1.007790 | 1.006410 | 1.005078 | 1.003565 |  | 111.18 | 111.65 | 112.21 | 112.89 | 113.47 |
| 0.1496 | 1.010847 | 1.009428 | 1.008040 | 1.006685 | 1.005152 |  | 111.28 | 111.72 | 112.25 | 112.98 | 113.61 |
|  |  |  |  |  |  |  |  |  |  |  |  |
| D-Fructose in aqueous solution of [Ch][Ace] (0.03 mol.kg^-1^) | | | | | | | | | | | |
| 0.0000 | 0.997253 | 0.995950 | 0.994695 | 0.993498 | 0.992379 |  |  |  |  |  |  |
| 0.0250 | 0.998983 | 0.997667 | 0.996400 | 0.995191 | 0.994061 |  | 110.89 | 111.47 | 112.01 | 112.55 | 113.05 |
| 0.0500 | 1.000700 | 0.999372 | 0.998095 | 0.996875 | 0.995736 |  | 110.90 | 111.46 | 111.96 | 112.48 | 112.93 |
| 0.0747 | 1.002396 | 1.001056 | 0.999768 | 0.998540 | 0.997392 |  | 110.89 | 111.45 | 111.95 | 112.42 | 112.87 |
| 0.1000 | 1.004121 | 1.002771 | 1.001471 | 1.000233 | 0.999080 |  | 110.87 | 111.40 | 111.91 | 112.37 | 112.77 |
| 0.1250 | 1.005812 | 1.004450 | 1.003139 | 1.001896 | 1.000737 |  | 110.85 | 111.38 | 111.89 | 112.32 | 112.70 |
| 0.1499 | 1.007489 | 1.006122 | 1.004796 | 1.003542 | 1.002386 |  | 110.84 | 111.32 | 111.86 | 112.30 | 112.60 |
|  |  |  |  |  |  |  |  |  |  |  |  |
| D-Fructose in aqueous solution of [Ch][Ace] (0.06 mol.kg^-1^) | | | | | | | | | | | |
| 0.0000 | 0.997641 | 0.996332 | 0.995069 | 0.993868 | 0.992732 |  |  |  |  |  |  |
| 0.0250 | 0.999369 | 0.998045 | 0.996771 | 0.995558 | 0.994409 |  | 110.95 | 111.61 | 112.11 | 112.65 | 113.23 |
| 0.0500 | 1.001089 | 0.999754 | 0.998470 | 0.997246 | 0.996085 |  | 110.92 | 111.50 | 111.98 | 112.49 | 113.05 |
| 0.0750 | 1.002798 | 1.001451 | 1.000155 | 0.998924 | 0.997752 |  | 110.90 | 111.47 | 111.96 | 112.42 | 112.96 |
| 0.0999 | 1.004499 | 1.003140 | 1.001831 | 1.000591 | 0.999415 |  | 110.88 | 111.44 | 111.96 | 112.41 | 112.87 |
| 0.1250 | 1.006197 | 1.004826 | 1.003507 | 1.002265 | 1.001076 |  | 110.88 | 111.43 | 111.94 | 112.33 | 112.81 |
| 0.1500 | 1.007880 | 1.006495 | 1.005166 | 1.003915 | 1.002728 |  | 110.85 | 111.42 | 111.92 | 112.32 | 112.71 |
|  |  |  |  |  |  |  |  |  |  |  |  |
| D-Fructose in aqueous solution of [Ch][Ace] (0.09 mol.kg^-1^) | | | | | | | | | | | |
| 0.0000 | 0.998026 | 0.996704 | 0.995435 | 0.994240 | 0.993113 |  |  |  |  |  |  |
| 0.0250 | 0.999729 | 0.998398 | 0.997118 | 0.995912 | 0.994776 |  | 111.96 | 112.38 | 112.88 | 113.38 | 113.79 |
| 0.0500 | 1.001432 | 1.000090 | 0.998797 | 0.997583 | 0.996442 |  | 111.75 | 112.21 | 112.75 | 113.19 | 113.53 |
| 0.0750 | 1.003134 | 1.001780 | 1.000472 | 0.999250 | 0.998100 |  | 111.54 | 112.03 | 112.61 | 113.03 | 113.40 |
| 0.1000 | 1.004840 | 1.003477 | 1.002153 | 1.000926 | 0.999762 |  | 111.37 | 111.84 | 112.45 | 112.83 | 113.26 |
| 0.1250 | 1.006536 | 1.005164 | 1.003824 | 1.002590 | 1.001413 |  | 111.20 | 111.67 | 112.30 | 112.67 | 113.13 |
| 0.1495 | 1.008211 | 1.006821 | 1.005476 | 1.004230 | 1.003035 |  | 111.00 | 111.52 | 112.09 | 112.49 | 113.00 |
|  |  |  |  |  |  |  |  |  |  |  |  |

^a^ The standard uncertainties for molality, temperature and pressure were *u* (*m*) *=* 0.001 mol kg^-1^, *u* (*T*) *=* 0.2K, *u* (*P*) *=* 10.5 hPa, respectively with level of confidence 0.95. The standard combined uncertainty for density and apparent molar volume were about, *u_c_* (*ρ*) *=* 0.06×10^-3^ g cm^-3^ and *u_c_*(*V_φ_*) = 5×10^-5^ m^3^ mol^-1^ (level of confidence 0.68), respectively.

**Table S2.** Standard apparent molar expansibility , , coefficient of thermal expansion, $\alpha$, Hepler's constant expansion, of D-Fructose in water and in the aqueous ILs solutions at *T* = (298.15 to 318.15) K and under atmospheric pressure of 0.0871 MPa.^a^

| *T/*K | *E*_ϕ_^0^/ m^3^·mol^-1^·K^-1^ | 10^3^/K^-1^ | 10^2^ |
| --- | --- | --- | --- |
| D-Fructose + water | | | |
| *m*_2_ = 0.000 mol·kg^-1^ | | | |
| 298.15 | 0.112 | 10.27 | -1.69 |
| 303.15 | 0.104 | 9.46 |
| 308.15 | 0.096 | 8.65 |
| 313.15 | 0.087 | 7.85 |
| 318.15 | 0.079 | 7.07 |
| D-Fructose in aqueous solutions of [Ch][Sal] | | | |
| *m*_2_ = 0.0300 mol·kg^-1^ | | | |
| 298.15 | 0.115 | 10.53 | 1.03 |
| 303.15 | 0.121 | 10.94 |
| 308.15 | 0.126 | 11.35 |
| 313.15 | 0.131 | 11.75 |
| 318.15 | 0.136 | 12.14 |
| *m*_2_ = 0.0600 mol·kg^-1^ | | | |
| 298.15 | 0.073 | 6.63 | 3.56 |
| 303.15 | 0.075 | 6.77 |
| 308.15 | 0.077 | 6.90 |
| 313.15 | 0.079 | 7.04 |
| 318.15 | 0.081 | 7.17 |
| *m*_2_ = 0.0900 mol·kg^-1^ | | | |
| 298.15 | 0.074 | 6.65 | 9.06 |
| 303.15 | 0.078 | 7.03 |
| 308.15 | 0.083 | 7.41 |
| 313.15 | 0.087 | 7.79 |
| 318.15 | 0.092 | 8.16 |
| D-Fructose in aqueous solutions of [Ch][For] | | | |
| *m*_2_ = 0.0300 mol·kg^-1^ | | | |
| 298.15 | 0.105 | 9.516 | -0.001 |
| 303.15 | 0.096 | 8.684 |
| 308.15 | 0.088 | 7.859 |
| 313.15 | 0.079 | 7.045 |
| 318.15 | 0.070 | 6.236 |
| *m*_2_ = 0.0600 mol·kg^-1^ | | | |
| 298.15 | 0.083 | 7.525 | -0.001 |
| 303.15 | 0.082 | 7.413 |
| 308.15 | 0.081 | 7.306 |
| 313.15 | 0.081 | 7.198 |
| 318.15 | 0.081 | 7.09 |
| *m*_2_ = 0.0900 mol·kg^-1^ | | | |
| 298.15 | 0.075 | 6.724 | 0.003 |
| 303.15 | 0.076 | 6.857 |
| 308.15 | 0.078 | 6.991 |
| 313.15 | 0.080 | 7.123 |
| 318.15 | 0.082 | 7.252 |
| D-Fructose in aqueous solutions of [Ch][Ace] | | | |
| *m*_2_ = 0.0300 mol·kg^-1^ | | | |
| 298.15 | 0.114 | 10.251 | -0.004 |
| 303.15 | 0.112 | 10.008 |
| 308.15 | 0.110 | 9.774 |
| 313.15 | 0.107 | 9.539 |
| 318.15 | 0.105 | 9.308 |
| *m*_2_ = 0.0600 mol·kg^-1^ | | | |
| 298.15 | 0.110 | 9.955 | 0.0003 |
| 303.15 | 0.112 | 10.055 |
| 308.15 | 0.114 | 10.166 |
| 313.15 | 0.116 | 10.268 |
| 303.15 | 0.112 | 10.055 |
|  | | | |
| *m*_2_ = 0.0900 mol·kg^-1^ | | | |
| 298.15 | 0.108 | 9.614 | -0.001 |
| 303.15 | 0.099 | 8.789 |
| 308.15 | 0.090 | 7.964 |
| 313.15 | 0.081 | 7.147 |
| 318.15 | 0.072 | 6.347 |

^a^ The standard uncertainties for molality, temperature and pressure were *u* (*m*) *=* 0.001 mol kg^-1^, *u* (*T*) *=* 0.2K, *u* (*P*) *=* 10.5 hPa, respectively with level of confidence 0.68.

**Table S3.** The values of speed of sound, *u*, and apparent molar isentropic compressibility, , for D-Fructose in the aqueous ILs solutions at different temperature and *P*= 0.0871 MPa. *^a^*

| *m* (mol·kg^-1^) | *u* (m·s^-1^) | | | | |  | 10^14^ *κ*_φ_ (m^3^·mol^-1^·Pa^-1^) | | | | |
| --- | --- | --- | --- | --- | --- | --- | --- | --- | --- | --- | --- |
| *T* (K) | 298.15 | 303.15 | 308.15 | 313.15 | 318.15 |  | 298.15 | 303.15 | 308.15 | 313.15 | 318.15 |
| D-Fructose in water | | | | | | | | | | | |
| 0.0000 | 1496.96 | 1509.44 | 1520.15 | 1529.23 | 1536.73 |  | - | - | - | - | - |
| 0.0250 | 1498.65 | 1511.04 | 1521.67 | 1530.66 | 1538.08 |  | -2.315 | -2.009 | -1.748 | -1.485 | -1.266 |
| 0.0500 | 1500.3 | 1512.62 | 1523.16 | 1532.06 | 1539.35 |  | -2.24 | -1.961 | -1.687 | -1.425 | -1.152 |
| 0.0750 | 1501.94 | 1514.14 | 1524.65 | 1533.44 | 1540.68 |  | -2.193 | -1.882 | -1.659 | -1.378 | -1.142 |
| 0.1000 | 1503.5 | 1515.67 | 1526.08 | 1534.82 | 1542 |  | -2.103 | -1.84 | -1.595 | -1.343 | -1.113 |
| 0.1250 | 1505.14 | 1517.26 | 1527.57 | 1536.17 | 1543.31 |  | -2.076 | -1.82 | -1.557 | -1.291 | -1.092 |
| 0.1500 | 1506.77 | 1518.78 | 1529.09 | 1537.63 | 1544.62 |  | -2.048 | -1.779 | -1.554 | -1.29 | -1.061 |
|  |  |  |  |  |  |  |  |  |  |  |  |
| D-Fructose in aqueous solutions of [Ch][Sal] (0.0300 mol·kg^-1^*)* | | | | | | | | | | | |
| 0.0000 | 1500.44 | 1512.58 | 1522.91 | 1531.79 | 1539.16 |  | - | - | - | - | - |
| 0.0249 | 1502.14 | 1514.2 | 1524.45 | 1533.26 | 1540.53 |  | -2.298 | -2.011 | -1.743 | -1.504 | -1.213 |
| 0.0501 | 1503.81 | 1515.82 | 1525.95 | 1534.66 | 1541.9 |  | -2.231 | -1.978 | -1.661 | -1.384 | -1.172 |
| 0.0747 | 1505.37 | 1517.33 | 1527.36 | 1536 | 1543.08 |  | -2.122 | -1.869 | -1.56 | -1.303 | -1.022 |
| 0.0998 | 1507.03 | 1518.79 | 1528.86 | 1537.37 | 1544.45 |  | -2.091 | -1.781 | -1.535 | -1.257 | -1.037 |
| 0.1249 | 1508.71 | 1520.35 | 1530.21 | 1538.65 | 1545.72 |  | -2.074 | -1.745 | -1.447 | -1.177 | -0.975 |
| 0.1468 | 1509.73 | 1521.45 | 1531.43 | 1539.85 | 1546.52 |  | -1.888 | -1.617 | -1.406 | -1.148 | -0.829 |
|  |  |  |  |  |  |  |  |  |  |  |  |
| D-Fructose in aqueous solutions of [Ch][Sal] (0.0600 mol·kg^-1^*)* | | | | | | | | | | | |
| 0.0000 | 1500.44 | 1512.58 | 1522.91 | 1531.79 | 1539.16 |  | - | - | - | - | - |
| 0.0249 | 1502.14 | 1514.2 | 1524.45 | 1533.26 | 1540.53 |  | -1.915 | -1.784 | -1.607 | -1.418 | -1.23 |
| 0.0501 | 1503.81 | 1515.82 | 1525.95 | 1534.66 | 1541.9 |  | -1.946 | -1.779 | -1.572 | -1.396 | -1.194 |
| 0.0747 | 1505.37 | 1517.33 | 1527.36 | 1536 | 1543.08 |  | -1.946 | -1.726 | -1.507 | -1.313 | -1.178 |
| 0.0998 | 1507.03 | 1518.79 | 1528.86 | 1537.37 | 1544.45 |  | -1.932 | -1.704 | -1.494 | -1.334 | -1.148 |
| 0.1249 | 1508.71 | 1520.35 | 1530.21 | 1538.65 | 1545.72 |  | -1.927 | -1.744 | -1.471 | -1.344 | -1.116 |
| 0.1468 | 1509.73 | 1521.45 | 1531.43 | 1539.85 | 1546.52 |  | -1.914 | -1.686 | -1.471 | -1.264 | -1.098 |
|  |  |  |  |  |  |  |  |  |  |  |  |
| D-Fructose in aqueous solutions of [Ch][Sal] (0.0900 mol·kg^-1^*)* | | | | | | | | | | | |
| 0.0000 | 1507.69 | 1519.43 | 1529.92 | 1539.23 | 1548.02 |  | - | - | - | - | - |
| 0.0250 | 1509.36 | 1521.07 | 1531.52 | 1540.78 | 1549.52 |  | -2.048 | -1.904 | -1.745 | -1.57 | -1.4 |
| 0.0502 | 1511.05 | 1522.7 | 1533.11 | 1542.28 | 1550.95 |  | -2.047 | -1.869 | -1.706 | -1.481 | -1.293 |
| 0.0751 | 1512.78 | 1524.28 | 1534.59 | 1543.71 | 1552.3 |  | -2.076 | -1.817 | -1.607 | -1.395 | -1.192 |
| 0.0998 | 1514.39 | 1525.84 | 1535.99 | 1545.1 | 1553.59 |  | -2.027 | -1.78 | -1.514 | -1.334 | -1.114 |
| 0.1248 | 1515.89 | 1527.2 | 1537.45 | 1546.36 | 1554.85 |  | -1.932 | -1.656 | -1.472 | -1.219 | -1.026 |
| 0.1496 | 1517.53 | 1528.78 | 1538.78 | 1547.75 | 1556.14 |  | -1.921 | -1.658 | -1.402 | -1.196 | -0.986 |
|  |  |  |  |  |  |  |  |  |  |  |  |
| D-Fructose in aqueous solutions of [Ch][For] (0.0300 mol·kg^-1^*)* | | | | | | | | | | | |
| 0.0000 | 1498.95 | 1511.21 | 1521.98 | 1531.16 | 1539.15 |  |  |  |  |  |  |
| 0.0252 | 1500.59 | 1512.76 | 1523.43 | 1532.56 | 1540.49 |  | -2.07 | -1.77 | -1.47 | -1.30 | -1.12 |
| 0.0499 | 1502.27 | 1514.33 | 1524.94 | 1533.93 | 1541.82 |  | -2.15 | -1.82 | -1.56 | -1.30 | -1.13 |
| 0.0753 | 1503.90 | 1515.84 | 1526.34 | 1535.37 | 1543.25 |  | -2.09 | -1.75 | -1.47 | -1.30 | -1.18 |
| 0.1000 | 1505.54 | 1517.45 | 1527.92 | 1536.82 | 1544.41 |  | -2.08 | -1.79 | -1.54 | -1.33 | -1.06 |
| 0.1249 | 1507.14 | 1518.93 | 1529.21 | 1538.12 | 1545.78 |  | -2.05 | -1.74 | -1.45 | -1.27 | -1.07 |
| 0.1499 | 1508.93 | 1520.61 | 1530.83 | 1539.47 | 1547.09 |  | -2.10 | -1.78 | -1.50 | -1.24 | -1.06 |
|  |  |  |  |  |  |  |  |  |  |  |  |
| D-Fructose in aqueous solutions of [Ch][For] (0.0600 mol·kg^-1^*)* | | | | | | | | | | | |
| 0.0000 | 1501.21 | 1513.38 | 1524.05 | 1533.03 | 1540.56 |  |  |  |  |  |  |
| 0.0252 | 1502.87 | 1514.97 | 1525.53 | 1534.42 | 1541.87 |  | -2.09 | -1.84 | -1.53 | -1.27 | -1.04 |
| 0.0501 | 1504.58 | 1516.55 | 1526.99 | 1535.80 | 1543.21 |  | -2.16 | -1.84 | -1.51 | -1.27 | -1.09 |
| 0.0752 | 1506.20 | 1518.03 | 1528.48 | 1537.22 | 1544.50 |  | -2.10 | -1.75 | -1.51 | -1.28 | -1.05 |
| 0.1001 | 1507.77 | 1519.61 | 1529.95 | 1538.59 | 1545.70 |  | -2.05 | -1.77 | -1.51 | -1.26 | -0.98 |
| 0.1253 | 1509.49 | 1521.13 | 1531.30 | 1539.87 | 1547.01 |  | -2.06 | -1.74 | -1.45 | -1.21 | -0.99 |
| 0.1501 | 1511.01 | 1522.76 | 1532.80 | 1541.13 | 1548.21 |  | -2.01 | -1.75 | -1.45 | -1.16 | -0.95 |
|  |  |  |  |  |  |  |  |  |  |  |  |
| D-Fructose in aqueous solutions of [Ch][For] (0.0900 mol·kg^-1^*)* | | | | | | | | | | | |
| 0.0000 | 1503.52 | 1515.45 | 1525.96 | 1534.86 | 1542.41 |  |  |  |  |  |  |
| 0.0252 | 1505.18 | 1517.07 | 1527.53 | 1536.36 | 1543.85 |  | -2.05 | -1.88 | -1.70 | -1.48 | -1.31 |
| 0.0503 | 1506.86 | 1518.67 | 1529.08 | 1537.84 | 1545.25 |  | -2.07 | -1.85 | -1.68 | -1.46 | -1.26 |
| 0.0748 | 1508.42 | 1520.19 | 1530.55 | 1539.27 | 1546.63 |  | -2.00 | -1.80 | -1.61 | -1.43 | -1.24 |
| 0.1001 | 1510.10 | 1521.79 | 1532.00 | 1540.67 | 1548.10 |  | -2.00 | -1.79 | -1.56 | -1.38 | -1.26 |
| 0.1247 | 1511.63 | 1523.25 | 1533.55 | 1542.07 | 1549.32 |  | -1.95 | -1.73 | -1.57 | -1.35 | -1.17 |
| 0.1498 | 1513.24 | 1524.82 | 1535.06 | 1543.62 | 1550.70 |  | -1.93 | -1.72 | -1.55 | -1.37 | -1.16 |
|  |  |  |  |  |  |  |  |  |  |  |  |
| D-Fructose in aqueous solutions of [Ch][Ace] (0.0300 mol·kg^-1^*)* | | | | | | | | | | | |
| 0.0000 | 1500.16 | 1512.15 | 1522.62 | 1531.41 | 1538.75 |  |  |  |  |  |  |
| 0.0250 | 1501.82 | 1513.72 | 1524.10 | 1532.79 | 1540.07 |  | -2.11 | -1.81 | -1.52 | -1.23 | -1.04 |
| 0.0500 | 1503.45 | 1515.28 | 1525.54 | 1534.11 | 1541.33 |  | -2.07 | -1.79 | -1.48 | -1.17 | -0.98 |
| 0.0747 | 1505.05 | 1516.80 | 1526.99 | 1535.46 | 1542.60 |  | -2.04 | -1.76 | -1.47 | -1.17 | -0.98 |
| 0.1000 | 1506.65 | 1518.34 | 1528.45 | 1536.80 | 1543.90 |  | -2.00 | -1.74 | -1.46 | -1.15 | -0.98 |
| 0.1250 | 1508.24 | 1519.88 | 1529.91 | 1538.10 | 1545.10 |  | -1.98 | -1.73 | -1.46 | -1.13 | -0.94 |
| 0.1499 | 1509.80 | 1521.40 | 1531.34 | 1539.42 | 1546.31 |  | -1.96 | -1.73 | -1.45 | -1.13 | -0.92 |
|  |  |  |  |  |  |  |  |  |  |  |  |
| D-Fructose in aqueous solutions of [Ch][Ace] (0.0600 mol·kg^-1^*)* | | | | | | | | | | | |
| 0.0000 | 1503.46 | 1515.37 | 1525.59 | 1534.25 | 1541.66 |  |  |  |  |  |  |
| 0.0250 | 1505.10 | 1516.94 | 1527.08 | 1535.61 | 1542.99 |  | -2.04 | -1.78 | -1.52 | -1.16 | -1.04 |
| 0.0500 | 1506.73 | 1518.44 | 1528.51 | 1536.96 | 1544.21 |  | -2.02 | -1.70 | -1.46 | -1.16 | -0.92 |
| 0.0750 | 1508.37 | 1519.97 | 1529.93 | 1538.30 | 1545.47 |  | -2.02 | -1.69 | -1.42 | -1.15 | -0.92 |
| 0.0999 | 1509.98 | 1521.50 | 1531.38 | 1539.66 | 1546.81 |  | -2.00 | -1.69 | -1.42 | -1.15 | -0.96 |
| 0.1250 | 1511.58 | 1523.00 | 1532.84 | 1540.97 | 1548.01 |  | -1.98 | -1.66 | -1.42 | -1.13 | -0.92 |
| 0.1500 | 1513.15 | 1524.53 | 1534.04 | 1542.28 | 1549.25 |  | -1.95 | -1.66 | -1.32 | -1.12 | -0.91 |
|  |  |  |  |  |  |  |  |  |  |  |  |
| D-Fructose in aqueous solutions of [Ch][Ace] (0.0900 mol·kg^-1^*)* | | | | | | | | | | | |
| 0.0000 | 1506.88 | 1518.61 | 1528.73 | 1537.26 | 1544.30 |  |  |  |  |  |  |
| 0.0250 | 1508.51 | 1520.16 | 1530.19 | 1538.61 | 1545.59 |  | -1.90 | -1.64 | -1.37 | -1.06 | -0.89 |
| 0.0500 | 1510.12 | 1521.65 | 1531.59 | 1539.95 | 1546.85 |  | -1.89 | -1.58 | -1.31 | -1.06 | -0.87 |
| 0.0750 | 1511.71 | 1523.15 | 1532.99 | 1541.26 | 1548.10 |  | -1.88 | -1.58 | -1.29 | -1.05 | -0.86 |
| 0.1000 | 1513.28 | 1524.64 | 1534.44 | 1542.57 | 1549.34 |  | -1.86 | -1.57 | -1.31 | -1.04 | -0.85 |
| 0.1250 | 1514.92 | 1526.10 | 1535.83 | 1543.87 | 1550.55 |  | -1.89 | -1.56 | -1.31 | -1.04 | -0.84 |
| 0.1495 | 1516.48 | 1527.54 | 1537.18 | 1545.13 | 1551.75 |  | -1.89 | -1.56 | -1.30 | -1.04 | -0.83 |
|  |  |  |  |  |  |  |  |  |  |  |  |

^a^ The standard uncertainties for molality, temperature and pressure were *u* (*m*) *=* 0.001 mol kg^-1^, *u* (*T*) *=* 0.2K, *u* (*P*) *=* 10.5 hPa, respectively with level of confidence 0.95. The standard combined uncertainty for speed of sound and apparent molar compressibility were estimated to be, *u_c_* (*u*) = 1.5 m s^-1^ and *u_c_*(*κ_φ_*) = 3.10^-13^ m^3^ mol·Pa^-1^  (level of confidence 0.68), respectively.

**Table S4.** The viscosity (*η*) values of D-Fructose in water and aqueous ILs solutions at 288.15 - 318.15 K and 0.0871 MPa.

| *m* (mol·kg^-1^) |  | | 10^-3^*η* (m·Pa·s) | | | |
| --- | --- | --- | --- | --- | --- | --- |
| *T (K)* | 298.15 | | 303.15 | 308.15 | 313.15 | 318.15 |
|  | | D-Fructose in aqueous solutions of [Ch][Sal] (0.0300 mol·kg^-1^*)* | | | | |
| 0.0253 | 0.913 | | 0.82 | 0.742 | 0.677 | 0.644 |
| 0.0496 | 0.922 | | 0.828 | 0.749 | 0.683 | 0.644 |
| 0.0757 | 0.931 | | 0.835 | 0.756 | 0.689 | 0.644 |
| 0.1020 | 0.94 | | 0.843 | 0.763 | 0.695 | 0.644 |
| 0.1279 | 0.949 | | 0.851 | 0.77 | 0.701 | 0.644 |
| 0.1543 | 0.958 | | 0.859 | 0.777 | 0.707 | 0.644 |
|  |  | |  |  |  |  |
|  | | D-Fructose in aqueous solutions of [Ch][Sal] (0.0600 mol·kg^-1^*)* | | | | |
| 0.0249 | 0.926 | | 0.838 | 0.76 | 0.686 | 0.627 |
| 0.0505 | 0.939 | | 0.848 | 0.768 | 0.7 | 0.635 |
| 0.0764 | 0.951 | | 0.858 | 0.777 | 0.713 | 0.644 |
| 0.1018 | 0.963 | | 0.868 | 0.797 | 0.728 | 0.658 |
| 0.1281 | 0.976 | | 0.878 | 0.803 | 0.734 | 0.671 |
| 0.1533 | 0.988 | | 0.888 | 0.816 | 0.755 | 0.679 |
|  |  | |  |  |  |  |
|  | | D-Fructose in aqueous solutions of [Ch][Sal] (0.0900 mol·kg^-1^*)* | | | | |
| 0.0251 | 0.937 | | 0.839 | 0.767 | 0.706 | 0.635 |
| 0.0506 | 0.948 | | 0.85 | 0.775 | 0.712 | 0.645 |
| 0.0760 | 0.958 | | 0.861 | 0.783 | 0.718 | 0.655 |
| 0.1019 | 0.969 | | 0.872 | 0.791 | 0.724 | 0.66 |
| 0.1281 | 0.98 | | 0.882 | 0.799 | 0.731 | 0.665 |
| 0.1531 | 0.994 | | 0.889 | 0.806 | 0.736 | 0.669 |
|  |  | |  |  |  |  |
|  | | D-Fructose in aqueous solutions of [Ch][For] (0.0300 mol·kg^-1^*)* | | | | |
| 0.0252 | 0.907 | | 0.815 | 0.737 | 0.673 | 0.616 |
| 0.0505 | 0.916 | | 0.823 | 0.746 | 0.683 | 0.624 |
| 0.0761 | 0.926 | | 0.838 | 0.753 | 0.700 | 0.639 |
| 0.1020 | 0.936 | | 0.854 | 0.778 | 0.714 | 0.649 |
| 0.1251 | 0.947 | | 0.869 | 0.786 | 0.729 | 0.661 |
| 0.1546 | 0.956 | | 0.885 | 0.804 | 0.743 | 0.672 |
|  |  | |  |  |  |  |
|  | | D-Fructose in aqueous solutions of [Ch][For] (0.0600 mol·kg-1) | | | | |
| 0.0251 | 0.916 | | 0.825 | 0.745 | 0.685 | 0.609 |
| 0.0505 | 0.926 | | 0.842 | 0.763 | 0.699 | 0.622 |
| 0.0761 | 0.938 | | 0.857 | 0.777 | 0.712 | 0.636 |
| 0.1018 | 0.95 | | 0.873 | 0.791 | 0.727 | 0.654 |
| 0.1281 | 0.963 | | 0.886 | 0.81 | 0.741 | 0.671 |
| 0.1543 | 0.975 | | 0.9 | 0.828 | 0.755 | 0.684 |
|  |  | |  |  |  |  |
|  | | D-Fructose in aqueous solutions of [Ch][For] (0.0900 mol·kg-1) | | | | |
| 0.0251 | 0.922 | | 0.848 | 0.776 | 0.701 | 0.627 |
| 0.0505 | 0.935 | | 0.861 | 0.787 | 0.717 | 0.644 |
| 0.0758 | 0.952 | | 0.880 | 0.807 | 0.739 | 0.665 |
| 0.1018 | 0.969 | | 0.896 | 0.824 | 0.758 | 0.685 |
| 0.1281 | 0.985 | | 0.914 | 0.840 | 0.774 | 0.702 |
| 0.1542 | 1.002 | | 0.931 | 0.861 | 0.796 | 0.720 |
|  |  | |  |  |  |  |
|  | | D-Fructose in aqueous solutions of [Ch][Ace] (0.0300 mol·kg-1) | | | | |
| 0.0252 | 0.907 | | 0.815 | 0.737 | 0.673 | 0.619 |
| 0.0505 | 0.916 | | 0.823 | 0.746 | 0.683 | 0.624 |
| 0.0761 | 0.926 | | 0.838 | 0.753 | 0.7 | 0.636 |
| 0.102 | 0.936 | | 0.853 | 0.768 | 0.71 | 0.649 |
| 0.1251 | 0.947 | | 0.864 | 0.776 | 0.722 | 0.664 |
| 0.1546 | 0.956 | | 0.876 | 0.794 | 0.745 | 0.677 |
|  |  | |  |  |  |  |
|  | | D-Fructose in aqueous solutions of [Ch][Ace] (0.0600 mol·kg-1) | | | | |
| 0.0251 | 0.916 | | 0.825 | 0.745 | 0.685 | 0.609 |
| 0.0505 | 0.926 | | 0.842 | 0.763 | 0.699 | 0.622 |
| 0.0761 | 0.938 | | 0.857 | 0.777 | 0.712 | 0.636 |
| 0.1018 | 0.950 | | 0.873 | 0.791 | 0.727 | 0.654 |
| 0.1281 | 0.963 | | 0.886 | 0.810 | 0.741 | 0.671 |
| 0.1543 | 0.975 | | 0.900 | 0.828 | 0.755 | 0.684 |
|  |  | |  |  |  |  |
|  | | D-Fructose in aqueous solutions of [Ch][Ace] (0.0900 mol·kg-1) | | | | |
| 0.0251 | 0.922 | | 0.848 | 0.776 | 0.701 | 0.627 |
| 0.0505 | 0.935 | | 0.861 | 0.787 | 0.717 | 0.644 |
| 0.0758 | 0.952 | | 0.88 | 0.807 | 0.739 | 0.665 |
| 0.1018 | 0.969 | | 0.896 | 0.824 | 0.758 | 0.685 |
| 0.1281 | 0.985 | | 0.914 | 0.84 | 0.774 | 0.702 |
| 0.1542 | 1.002 | | 0.931 | 0.861 | 0.796 | 0.720 |
|  |  | |  |  |  |  |

^a^The standard uncertainties for molality, temperature and pressure were *u* (*m*) *=* 0.001 mol kg^-1^, *u* (*T*) *=* 0.2K, *u* (*P*) *=* 10.5 hPa, respectively with level of confidence 0.95. The standard combined uncertainty for viscosity was about, *u_c_* (*η*) = 0.02 m.Pa.s (level of confidence 0.68).

**Table S5.** Molar conductivities,$\Lambda$, of ILs in various concentrations of aqueous D-Fructose solutions at 298.15 K.

| *m*_fructose_ (mol·kg^-1^) | | | | | | | | | | | |
| --- | --- | --- | --- | --- | --- | --- | --- | --- | --- | --- | --- |
| 0 | | | 0.0499 | | | 0.0998 | | | 0.1501 | | |
| *C* | *κ* | *Λ* | *C* | *κ* | *Λ* | *C* | *κ* | *Λ* | *C* | *κ* | *Λ* |
| (mol.m^-3^) | (μS.cm^-1^) | (S.cm^2^.mol^-1^) | (mol.m^-3^) | (μS.cm^-1^) | (S.cm^2^.mol^-1^) | (mol.m^-3^) | (μS.cm^-1^) | (S.cm^2^.mol^-1^) | (mol.m^-3^) | (μS.cm^-1^) | (S.cm^2^.mol^-1^) |
| [Ch][Sal] | | | | | | | | | | | |
| 0.5557 | 37.49 | 67.458 | 0.4587 | 30.616 | 66.74 | 0.4734 | 30.939 | 65.355 | 0.1995 | 12.713 | 63.71 |
| 0.7441 | 49.68 | 66.769 | 0.6652 | 43.776 | 65.812 | 0.7406 | 47.489 | 64.125 | 0.4228 | 26.493 | 62.654 |
| 0.9186 | 60.71 | 66.09 | 0.8762 | 57.146 | 65.221 | 0.9937 | 63.059 | 63.46 | 0.6366 | 39.383 | 61.86 |
| 1.0748 | 70.6 | 65.689 | 1.0872 | 69.996 | 64.382 | 1.2702 | 79.919 | 62.918 | 0.8599 | 52.433 | 60.972 |
| 1.2585 | 81.81 | 65.007 | 1.3579 | 86.066 | 63.384 | 1.5421 | 95.719 | 62.072 | 1.1165 | 67.043 | 60.047 |
| 1.456 | 93.82 | 64.438 | 1.5551 | 97.756 | 62.861 | 1.8186 | 111.409 | 61.26 | 1.3351 | 79.383 | 59.46 |
| 1.5846 | 101.79 | 64.238 | 1.8074 | 111.596 | 61.743 | 2.0905 | 126.509 | 60.517 | 1.5679 | 92.013 | 58.687 |
| 1.7637 | 111.99 | 63.497 | 2.046 | 124.796 | 60.996 | 2.3342 | 140.009 | 59.982 | 1.7722 | 103.053 | 58.151 |
| 1.9245 | 121.49 | 63.130 | 2.2662 | 137.296 | 60.585 | 2.6295 | 156.309 | 59.445 | 1.9907 | 114.653 | 57.594 |
| 1.9933 | 125.69 | 63.055 | 2.5231 | 151.196 | 59.926 |  |  |  | 2.2568 | 128.353 | 56.875 |
|  | | | | | | | | | | | |
| [Ch][For] | | | | | | | | | | | |
| 0.3039 | 21.987 | 73.439 | 0.5763 | 71.216 | 71.216 | 0.2646 | 18.094 | 68.39 | 0.3678 | 21.743 | 59.120 |
| 0.673 | 48.217 | 72.143 | 0.8982 | 69.641 | 69.641 | 0.6577 | 43.394 | 65.983 | 0.8275 | 46.733 | 56.475 |
| 1.0131 | 71.907 | 71.274 | 1.2424 | 68.388 | 68.388 | 1.0129 | 63.344 | 62.535 | 1.2566 | 68.883 | 54.819 |
| 1.2302 | 86.937 | 70.666 | 1.5568 | 67.162 | 67.162 | 1.3985 | 83.094 | 59.418 | 1.7163 | 92.203 | 53.722 |
| 1.5848 | 110.807 | 69.917 | 1.9086 | 65.996 | 65.996 | 1.7311 | 99.294 | 57.360 | 2.1913 | 114.733 | 52.358 |
| 1.8816 | 130.407 | 69.308 | 2.3053 | 64.834 | 64.834 | 2.0939 | 116.294 | 55.539 | 2.6434 | 135.733 | 51.348 |
| 2.3447 | 160.507 | 68.455 | 2.5972 | 63.938 | 63.938 | 2.555 | 135.394 | 52.991 | 3.0954 | 155.933 | 50.375 |
|  | | | | | | | | | | | |
| [Ch][Ace] | | | | | | | | | | | |
| 0.2574 | 15.461 | 58.006 | 0.8461 | 43.718 | 51.672 | 0.7301 | 35.058 | 48.021 | 0.8183 | 38.508 | 47.060 |
| 0.4591 | 26.561 | 57.449 | 1.2460 | 61.958 | 49.724 | 1.064 | 50.398 | 47.365 | 1.2195 | 56.138 | 46.032 |
| 0.6887 | 38.561 | 55.990 | 1.6152 | 78.798 | 48.784 | 1.4368 | 66.468 | 46.26 | 1.5579 | 70.668 | 45.362 |
| 0.9113 | 49.711 | 54.548 | 2.0229 | 95.778 | 47.347 | 1.8019 | 81.478 | 45.219 | 1.9355 | 86.798 | 44.844 |
| 1.1896 | 62.991 | 52.951 | 2.4075 | 111.578 | 46.347 | 2.1436 | 95.358 | 44.485 | 2.3053 | 101.898 | 44.201 |
| 1.5444 | 79.021 | 51.166 | 2.7767 | 126.578 | 45.587 | 2.4775 | 108.658 | 43.857 | 2.6751 | 116.898 | 43.698 |
| 1.7809 | 89.641 | 50.334 | 3.1305 | 139.278 | 44.491 | 2.8348 | 122.858 | 43.339 | 3.0371 | 131.098 | 43.166 |
| 2.0453 | 101.041 | 49.402 | 3.5074 | 152.678 | 43.531 | 3.1532 | 134.358 | 42.609 | 3.399 | 145.198 | 42.718 |
| 2.3862 | 115.641 | 48.463 | 3.9150 | 166.578 | 42.549 | 3.5804 | 150.058 | 41.911 | 3.7609 | 158.898 | 42.250 |
| 2.6853 | 127.541 | 47.496 |  |  |  | 3.9687 | 164.358 | 41.413 | 4.1386 | 172.898 | 41.777 |
| 3.0470 | 141.841 | 46.550 |  |  |  | 4.2949 | 176.158 | 41.015 |  |  |  |
| 3.4714 | 157.841 | 45.469 |  |  |  |  |  |  |  |  |  |
| 3.9375 | 174.941 | 44.429 |  |  |  |  |  |  |  |  |  |
|  |  |  |  |  |  |  |  |  |  |  |  |

^a^The standard uncertainties for molality and temperature were *u* (*C*) *=* 0.001 mol m^-3^ and *u* (*T*) *=* 0.5 K, respectively with level of confidence 0.95. The standard combined uncertainty for conductance and molar conductivity were about, *u_c_* (*κ*) =0.5 μS.cm^-1^ and *u_c_*(*Λ*) = 0.7 μS.cm^2^.mol^-1^ (level of confidence 0.68), respectively.

**Table S6.** Hydration numbers, $n_{H}$, of D-fructose in water and in various aqueous choline based ILs solutions at temperatures, *T* = (293.15–318.15) K.

| *m* (mol·kg^-1^) | T(K) | | | | |
| --- | --- | --- | --- | --- | --- |
|  | 298.15 | 303.15 | 308.15 | 313.15 | 318.15 |
|  | $n_{H}$ | | | | |
| D-fructose in water | | | | | |
| 0.0000 | 2.413 | 2.07 | 1.743 | 1.490 | 1.306 |
| D-Fructose in aqueous solutions of [Ch][Sal] | | | | | |
| 0.0300 | 2.374 | 2.009 | 1.661 | 1.373 | 1.147 |
| 0.0600 | 2.031 | 1.738 | 1.486 | 1.281 | 1.104 |
| 0.0900 | 1.995 | 1.724 | 1.451 | 1.238 | 1.063 |
| D-Fructose in aqueous solutions of [Ch][For] | | | | | |
| 0.0300 | 2.109 | 1.789 | 1.498 | 1.287 | 1.112 |
| 0.0600 | 2.051 | 1.737 | 1.472 | 1.261 | 1.0900 |
| 0.0900 | 1.993 | 1.701 | 1.443 | 1.239 | 1.068 |
| D-Fructose in aqueous solutions of [Ch][Ace] | | | | | |
| 0.0300 | 1.971 | 1.637 | 1.349 | 1.112 | 0.925 |
| 0.0600 | 1.959 | 1.615 | 1.336 | 1.097 | 0.892 |
| 0.0900 | 1.604 | 1.349 | 1.0900 | 0.889 | 0.762 |

**Table S7.** The values of apparent specific volume, *ASV,* and apparent specific isentropic compressibility, *ASIC,* values for D-fructose in water and aqueous ILs solutions at *T* = (288.15 to 318.15) K.

| *m* (mol·kg^-1^) | *ASV* (cm^3^·g^-1^) | | | | |  | 10^14^ *ASIC* (m^3^·g^-1^·Pa^-1^) | | | | |
| --- | --- | --- | --- | --- | --- | --- | --- | --- | --- | --- | --- |
| *T* (K) | 298.15 | 303.15 | 308.15 | 313.15 | 318.15 |  | 298.15 | 303.15 | 308.15 | 313.15 | 318.15 |
| D-Fructose in water | | | | | | | | | | | |
| 0.0000 | - | - | - | - | - |  | - | - | - | - | - |
| 0.0250 | 0.611 | 0.614 | 0.617 | 0.619 | 0.622 |  | -0.013 | -0.011 | -0.010 | -0.008 | -0.007 |
| 0.0500 | 0.612 | 0.615 | 0.618 | 0.621 | 0.623 |  | -0.012 | -0.011 | -0.009 | -0.008 | -0.006 |
| 0.0750 | 0.613 | 0.616 | 0.618 | 0.621 | 0.624 |  | -0.012 | -0.010 | -0.009 | -0.008 | -0.006 |
| 0.1000 | 0.614 | 0.616 | 0.619 | 0.622 | 0.625 |  | -0.012 | -0.010 | -0.009 | -0.007 | -0.006 |
| 0.1250 | 0.614 | 0.617 | 0.62 | 0.623 | 0.625 |  | -0.012 | -0.010 | -0.009 | -0.007 | -0.006 |
| 0.1500 | 0.615 | 0.618 | 0.62 | 0.623 | 0.626 |  | -0.011 | -0.010 | -0.009 | -0.007 | -0.006 |
|  |  |  |  |  |  |  |  |  |  |  |  |
| D-Fructose in aqueous solutions of [Ch][Sal] (0.0300 mol·kg^-1^*)* | | | | | | | | | | | |
| 0.0000 | - | - | - | - | - |  | - | - | - | - | - |
| 0.0249 | 0.610 | 0.613 | 0.616 | 0.620 | 0.623 |  | -0.013 | -0.011 | -0.010 | -0.008 | -0.007 |
| 0.0501 | 0.610 | 0.614 | 0.617 | 0.622 | 0.625 |  | -0.012 | -0.011 | -0.009 | -0.008 | -0.007 |
| 0.0747 | 0.612 | 0.616 | 0.619 | 0.623 | 0.626 |  | -0.012 | -0.010 | -0.009 | -0.007 | -0.006 |
| 0.0998 | 0.613 | 0.616 | 0.621 | 0.624 | 0.627 |  | -0.012 | -0.010 | -0.009 | -0.007 | -0.006 |
| 0.1249 | 0.614 | 0.618 | 0.621 | 0.625 | 0.629 |  | -0.012 | -0.010 | -0.008 | -0.007 | -0.005 |
| 0.1468 | 0.615 | 0.619 | 0.623 | 0.628 | 0.631 |  | -0.01 | -0.009 | -0.008 | -0.006 | -0.005 |
|  |  |  |  |  |  |  |  |  |  |  |  |
| D-Fructose in aqueous solutions of [Ch][Sal] (0.0600 mol·kg^-1^*)* | | | | | | | | | | | |
| 0.0000 | - | - | - | - | - |  | - | - | - | - | - |
| 0.0249 | 0.615 | 0.616 | 0.619 | 0.621 | 0.622 |  | -0.011 | -0.010 | -0.009 | -0.008 | -0.007 |
| 0.0501 | 0.615 | 0.617 | 0.619 | 0.621 | 0.623 |  | -0.011 | -0.010 | -0.009 | -0.008 | -0.007 |
| 0.0747 | 0.616 | 0.618 | 0.619 | 0.621 | 0.623 |  | -0.011 | -0.010 | -0.008 | -0.007 | -0.007 |
| 0.0998 | 0.616 | 0.618 | 0.62 | 0.622 | 0.624 |  | -0.011 | -0.009 | -0.008 | -0.007 | -0.006 |
| 0.1249 | 0.616 | 0.618 | 0.62 | 0.622 | 0.624 |  | -0.011 | -0.010 | -0.008 | -0.007 | -0.006 |
| 0.1468 | 0.616 | 0.618 | 0.62 | 0.622 | 0.624 |  | -0.011 | -0.009 | -0.008 | -0.007 | -0.006 |
|  |  |  |  |  |  |  |  |  |  |  |  |
| D-Fructose in aqueous solutions of [Ch][Sal] (0.0900 mol·kg^-1^*)* | | | | | | | | | | | |
| 0.0000 | - | - | - | - | - |  | - | - | - | - | - |
| 0.0250 | 0.616 | 0.618 | 0.62 | 0.622 | 0.624 |  | -0.011 | -0.011 | -0.010 | -0.009 | -0.008 |
| 0.0502 | 0.616 | 0.618 | 0.62 | 0.622 | 0.625 |  | -0.011 | -0.010 | -0.009 | -0.008 | -0.007 |
| 0.0751 | 0.616 | 0.619 | 0.62 | 0.623 | 0.625 |  | -0.012 | -0.010 | -0.009 | -0.008 | -0.007 |
| 0.0998 | 0.616 | 0.619 | 0.621 | 0.623 | 0.625 |  | -0.011 | -0.010 | -0.008 | -0.007 | -0.006 |
| 0.1248 | 0.617 | 0.619 | 0.621 | 0.623 | 0.627 |  | -0.011 | -0.009 | -0.008 | -0.007 | -0.006 |
| 0.1496 | 0.617 | 0.619 | 0.622 | 0.625 | 0.627 |  | -0.011 | -0.009 | -0.008 | -0.007 | -0.005 |
|  |  |  |  |  |  |  |  |  |  |  |  |
| D-Fructose in aqueous solutions of [Ch][For] (0.0300 mol·kg^-1^*)* | | | | | | | | | | | |
| 0.0000 | - | - | - | - | - |  | - | - | - | - | - |
| 0.0252 | 0.610 | 0.613 | 0.616 | 0.620 | 0.623 |  | -0.011 | -0.010 | -0.008 | -0.007 | -0.006 |
| 0.0499 | 0.610 | 0.614 | 0.617 | 0.621 | 0.625 |  | -0.012 | -0.010 | -0.009 | -0.007 | -0.006 |
| 0.0753 | 0.612 | 0.616 | 0.619 | 0.623 | 0.626 |  | -0.012 | -0.010 | -0.008 | -0.007 | -0.007 |
| 0.1000 | 0.613 | 0.616 | 0.621 | 0.624 | 0.627 |  | -0.012 | -0.010 | -0.009 | -0.007 | -0.006 |
| 0.1249 | 0.615 | 0.618 | 0.621 | 0.625 | 0.629 |  | -0.011 | -0.010 | -0.008 | -0.007 | -0.006 |
| 0.1499 | 0.615 | 0.619 | 0.622 | 0.626 | 0.629 |  | -0.012 | -0.010 | -0.008 | -0.007 | -0.006 |
|  |  |  |  |  |  |  |  |  |  |  |  |
| D-Fructose in aqueous solutions of [Ch][For] (0.0600 mol·kg^-1^*)* | | | | | | | | | | | |
| 0.0000 | - | - | - | - | - |  | - | - | - | - | - |
| 0.0252 | 0.615 | 0.617 | 0.620 | 0.622 | 0.624 |  | -0.012 | -0.010 | -0.008 | -0.007 | -0.006 |
| 0.0501 | 0.615 | 0.618 | 0.620 | 0.623 | 0.625 |  | -0.012 | -0.010 | -0.008 | -0.007 | -0.006 |
| 0.0752 | 0.616 | 0.618 | 0.620 | 0.624 | 0.627 |  | -0.012 | -0.010 | -0.008 | -0.007 | -0.006 |
| 0.1001 | 0.616 | 0.618 | 0.621 | 0.625 | 0.628 |  | -0.011 | -0.010 | -0.008 | -0.007 | -0.005 |
| 0.1253 | 0.616 | 0.619 | 0.622 | 0.626 | 0.629 |  | -0.011 | -0.010 | -0.008 | -0.007 | -0.005 |
| 0.1501 | 0.617 | 0.619 | 0.623 | 0.627 | 0.630 |  | -0.011 | -0.010 | -0.008 | -0.006 | -0.005 |
|  |  |  |  |  |  |  |  |  |  |  |  |
| D-Fructose in aqueous solutions of [Ch][For] (0.0900 mol·kg^-1^*)* | | | | | | | | | | | |
| 0.0000 | - | - | - | - | - |  | - | - | - | - | - |
| 0.0252 | 0.616 | 0.618 | 0.62 | 0.623 | 0.625 |  | -0.011 | -0.010 | -0.009 | -0.008 | -0.007 |
| 0.0503 | 0.616 | 0.618 | 0.621 | 0.624 | 0.626 |  | -0.011 | -0.010 | -0.009 | -0.008 | -0.007 |
| 0.0748 | 0.616 | 0.619 | 0.622 | 0.625 | 0.628 |  | -0.011 | -0.010 | -0.009 | -0.008 | -0.007 |
| 0.1001 | 0.617 | 0.619 | 0.622 | 0.625 | 0.628 |  | -0.011 | -0.010 | -0.009 | -0.008 | -0.007 |
| 0.1247 | 0.617 | 0.62 | 0.623 | 0.627 | 0.63 |  | -0.011 | -0.010 | -0.009 | -0.007 | -0.006 |
| 0.1498 | 0.618 | 0.62 | 0.623 | 0.627 | 0.631 |  | -0.011 | -0.010 | -0.009 | -0.008 | -0.006 |
|  |  |  |  |  |  |  |  |  |  |  |  |
| D-Fructose in aqueous solutions of [Ch][Ace] (0.0300 mol·kg^-1^*)* | | | | | | | | | | | |
| 0.0000 | - | - | - | - | - |  | - | - | - | - | - |
| 0.0250 | 0.616 | 0.619 | 0.622 | 0.625 | 0.627 |  | -0.012 | -0.010 | -0.008 | -0.007 | -0.006 |
| 0.0500 | 0.616 | 0.619 | 0.621 | 0.624 | 0.627 |  | -0.011 | -0.010 | -0.008 | -0.006 | -0.005 |
| 0.0747 | 0.616 | 0.619 | 0.621 | 0.624 | 0.626 |  | -0.011 | -0.010 | -0.008 | -0.006 | -0.005 |
| 0.1000 | 0.615 | 0.618 | 0.621 | 0.624 | 0.626 |  | -0.011 | -0.010 | -0.008 | -0.006 | -0.005 |
| 0.1250 | 0.615 | 0.618 | 0.621 | 0.623 | 0.626 |  | -0.011 | -0.010 | -0.008 | -0.006 | -0.005 |
| 0.1499 | 0.615 | 0.618 | 0.621 | 0.623 | 0.625 |  | -0.011 | -0.010 | -0.008 | -0.006 | -0.005 |
|  |  |  |  |  |  |  |  |  |  |  |  |
| D-Fructose in aqueous solutions of [Ch][Ace] (0.0600 mol·kg^-1^*)* | | | | | | | | | | | |
| 0.0000 | - | - | - | - | - |  | - | - | - | - | - |
| 0.0250 | 0.616 | 0.620 | 0.622 | 0.625 | 0.628 |  | -0.011 | -0.010 | -0.008 | -0.006 | -0.006 |
| 0.0500 | 0.616 | 0.619 | 0.622 | 0.624 | 0.627 |  | -0.011 | -0.009 | -0.008 | -0.006 | -0.005 |
| 0.0750 | 0.616 | 0.619 | 0.621 | 0.624 | 0.627 |  | -0.011 | -0.009 | -0.008 | -0.006 | -0.005 |
| 0.0999 | 0.615 | 0.619 | 0.621 | 0.624 | 0.626 |  | -0.011 | -0.009 | -0.008 | -0.006 | -0.005 |
| 0.1250 | 0.615 | 0.619 | 0.621 | 0.624 | 0.626 |  | -0.011 | -0.009 | -0.008 | -0.006 | -0.005 |
| 0.1500 | 0.615 | 0.618 | 0.621 | 0.623 | 0.626 |  | -0.011 | -0.009 | -0.007 | -0.006 | -0.005 |
|  |  |  |  |  |  |  |  |  |  |  |  |
| D-Fructose in aqueous solutions of [Ch][Ace] (0.0900 mol·kg^-1^*)* | | | | | | | | | | | |
| 0.0000 | - | - | - | - | - |  | - | - | - | - | - |
| 0.0250 | 0.621 | 0.624 | 0.627 | 0.629 | 0.632 |  | -0.011 | -0.009 | -0.008 | -0.006 | -0.005 |
| 0.0500 | 0.620 | 0.623 | 0.626 | 0.628 | 0.63 |  | -0.01 | -0.009 | -0.007 | -0.006 | -0.005 |
| 0.0750 | 0.619 | 0.622 | 0.625 | 0.627 | 0.629 |  | -0.01 | -0.009 | -0.007 | -0.006 | -0.005 |
| 0.1000 | 0.618 | 0.621 | 0.624 | 0.626 | 0.629 |  | -0.01 | -0.009 | -0.007 | -0.006 | -0.005 |
| 0.1250 | 0.617 | 0.62 | 0.623 | 0.625 | 0.628 |  | -0.01 | -0.009 | -0.007 | -0.006 | -0.005 |
| 0.1495 | 0.616 | 0.619 | 0.622 | 0.624 | 0.627 |  | -0.01 | -0.009 | -0.007 | -0.006 | -0.005 |
|  |  |  |  |  |  |  |  |  |  |  |  |


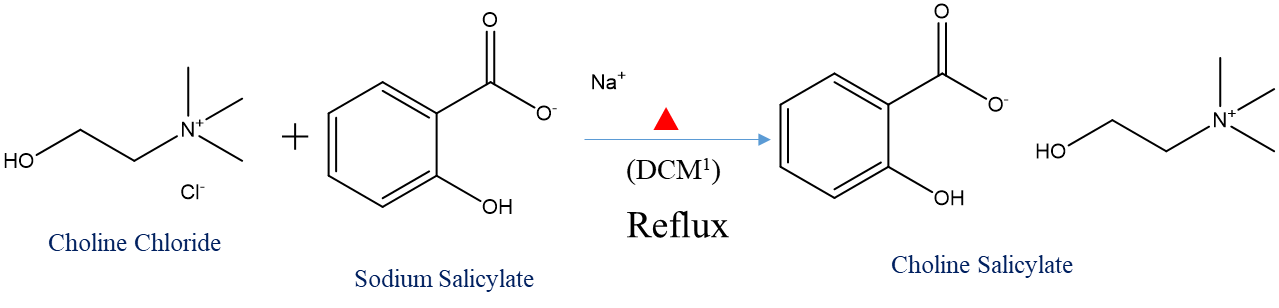


Fig S1. The synthesis plan of the [Ch][Sal] IL.

Figure S1, presents the synthesis route for [Ch][Sal] IL, the synthesis process is as follows: Equimolar quantities of choline chloride and sodium salicylate were introduced into a 250 mL round-bottom flask. Dichloromethane (DCM) was employed as a reaction medium, and approximately 80 mL was added to facilitate the reaction kinetics. The reaction vessel was immersed in an oil bath and subjected to vigorous magnetic stirring. The synthesis was conducted at 298.15 K under a neutral argon atmosphere for a duration of 72 hours to prevent oxidation of the target IL. Upon completion, the crude product was subjected to multiple centrifugation cycles to ensure complete removal of inorganic by-products, namely sodium chloride. Subsequently, the DCM solvent was evaporated under reduced pressure at 313.15 K using a rotary evaporator. To further purify the [Ch][Sal] IL, approximately 100 mL of anhydrous DCM was added and the mixture was vigorously agitated. This washing process effectively removed residual impurities and inorganic salts, resulting in a biphasic system. The lower, denser phase enriched in the desired IL was separated from the upper organic phase, which exhibited a turbid appearance due to the presence of impurities. This washing and separation procedure was reiterated until the upper phase attained clarity, indicating the removal of contaminants. The purified IL was finally dried under vacuum at room temperature in an argon-filled desiccator to minimize moisture content, as even trace amounts of water can significantly influence physical properties such as density, viscosity, and electrical conductivity.


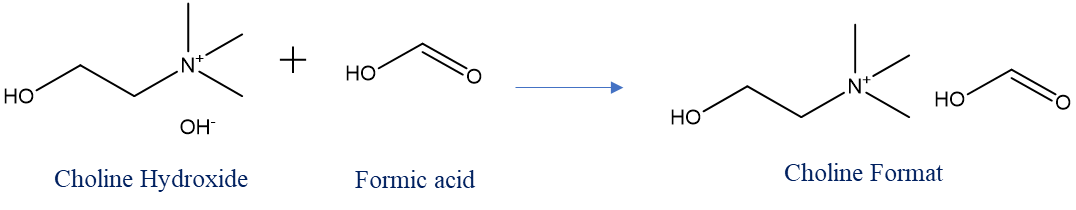


***Fig S2.*** The synthesis plan of the [Ch][For] IL.


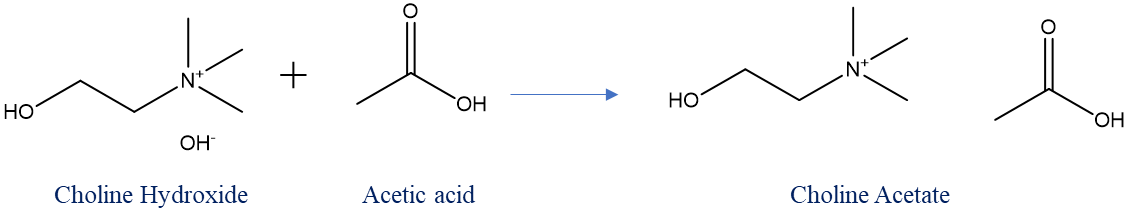


***Figure S3.*** The synthesis plan of the [Ch][Ace] IL.

[Ch][For] and [Ch][Ace] were synthesized through a neutralization process (Figs S4 and S5). Initially, choline hydroxide was produced by reacting choline chloride with potassium hydroxide in methanol under reflux conditions. After removing the methanol, the absence of chloride ions was confirmed. The resulting choline hydroxide solution was then titrated to determine its concentration. Subsequently, stoichiometric amounts of formic acid and acetic acid were added to the choline hydroxide solution and stirred at room temperature. The formed water was removed through vacuum distillation, and the crude product was washed with a methanol-acetonitrile mixture to purify the resulted ILs. The final products, [Ch][For] and [Ch][Ace], were obtained after removing the solvents. In order to confirm the purity and structural integrity of the synthesized ILs, further analysis using techniques such as FT-IR and FT-NMR spectroscopy was performed.


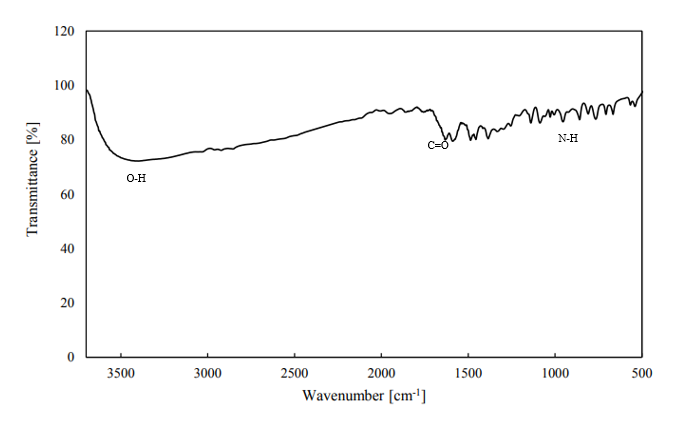


Fig S4. FT-IR spectrum of [Ch][Sal] IL.

FT-IR spectroscopy is a valuable technique for elucidating the functional groups present in a molecule. When applied to [Ch][Sal] IL (Fig S2), several key vibrational bands provide structural information. The IR spectrum exhibits a broad absorption band centered around 3500 cm^-1^, characteristic of hydroxyl (O-H) stretching vibrations. This indicates the presence of an alcohol or phenol group, likely associated with the salicylate moiety. Additionally, a sharp peak at 1756 cm^-1^ corresponds to the carbonyl (C=O) stretching vibration, confirming the presence of a carbonyl group within the salicylate structure. Multiple peaks in the region of 1579-63 cm^-1^ are attributed to the aromatic C-C stretching vibrations of the benzene ring, a fundamental component of the salicylate moiety. Furthermore, the FT-IR spectrum depicts absorption bands between 1207 and 1139 cm^-1^, which are probably assigned to C-N stretching vibrations. These bands arise from the nitrogen-containing functional groups present in both the choline and salicylate components of the IL.


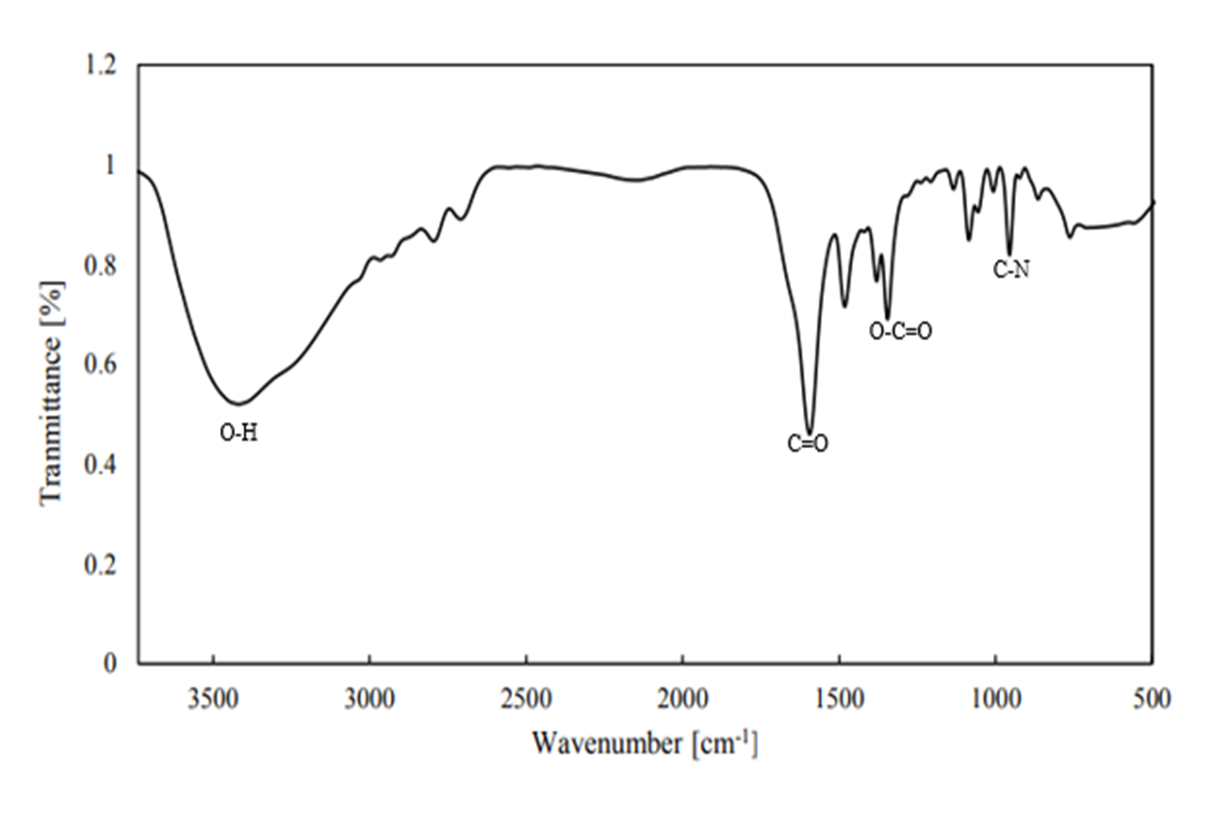


***Figure S5.*** FT-IR spectrum of [Ch][For] IL.

The FT-IR spectra analysis of [Ch][For] (Fig S6) provides valuable insights into its molecular structure. Key functional groups are identified through characteristic absorption bands. A prominent peak at 1594 cm^-1^ confirms the presence of a carbonyl (C=O) group, characteristic of the formate moiety. Additionally, the presence of a carboxylate group (COO^-^) is indicated by a band at 1346 cm^-1^, suggesting the formation of an ionic salt. Further supporting the formate structure by the C-O stretching vibration observed at 1083 cm^-1^. The presence of C-N bending vibrations around 956-65 cm^-1^ suggests the presence of nitrogen-containing functional groups within the choline cation.


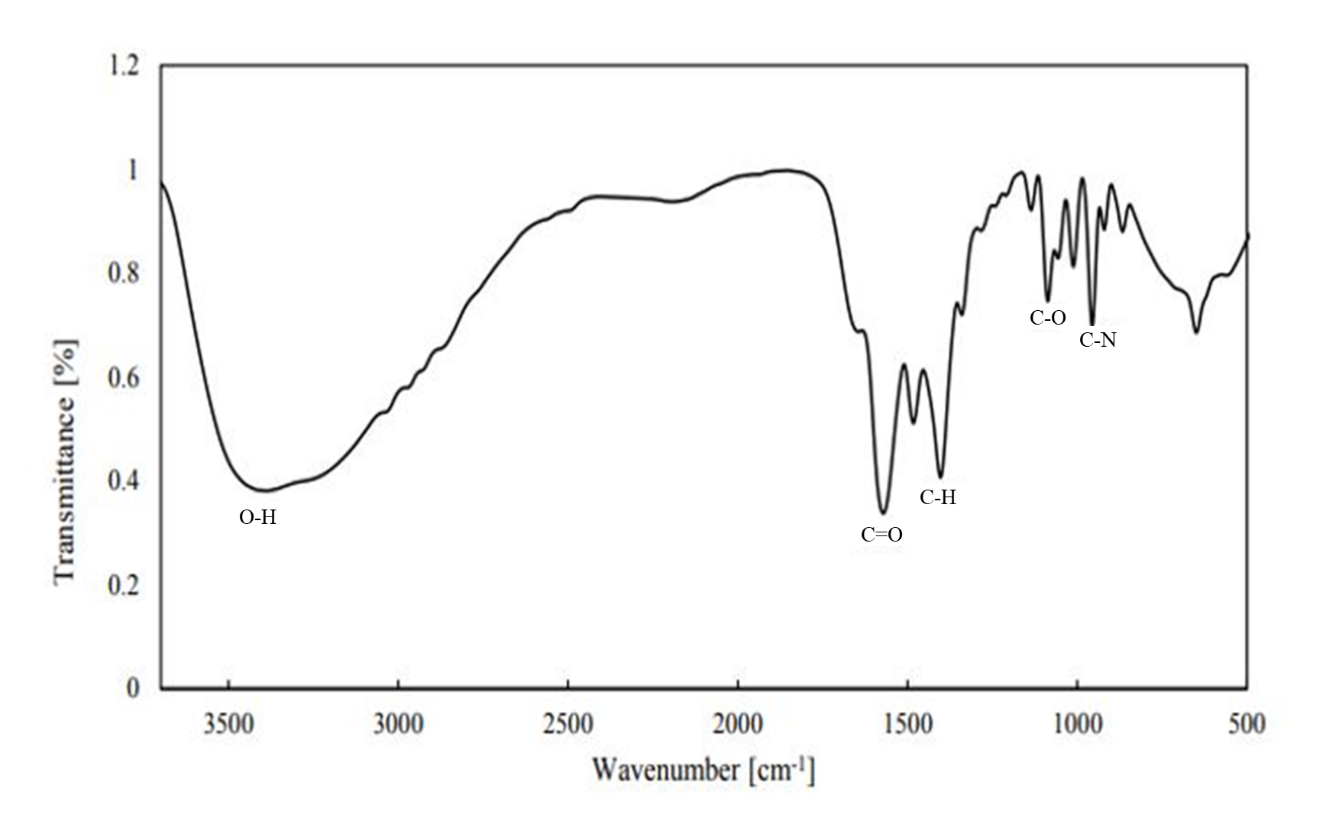


***Figure S6.*** FT-IR spectrum of [Ch][Ace] IL.

The FT-IR spectrum analysis of the [Ch][Ace] IL (Fig S8), provides valuable insights into its molecular structure. A broad peak around 3500 cm^-1^ indicates the presence of a hydroxyl (O-H) group. The carbonyl (C=O) stretching vibration is observed at 1087 cm^-1^, confirming the presence of the acetate group. Additionally, the C-H bending vibration at 1404 cm^-1^ suggests the presence of aliphatic groups. The N-H stretching vibration at 956 cm^-1^ indicates the presence of an amine group, likely associated with the choline cation.


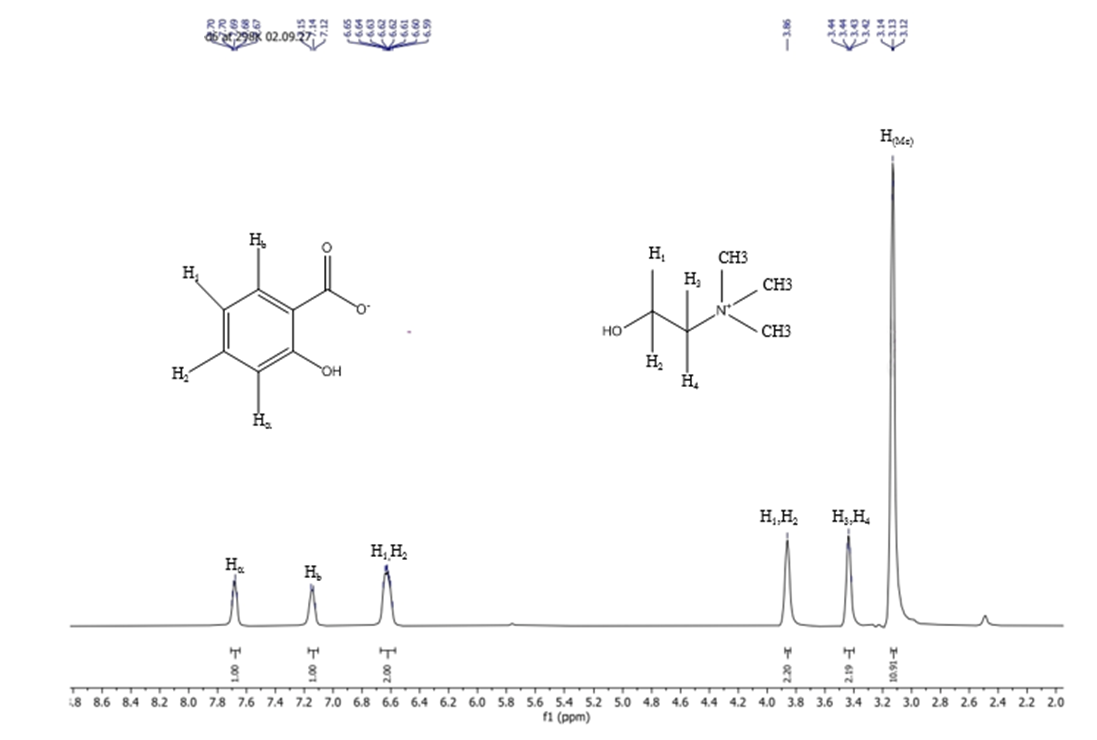


***Fig S7*.** ^1^H-NMR (400 MHz, DMSO) spectrum of the [Ch][Sal] IL.

A Bruker Avance-400 NMR spectrometer was employed for the ^1^H-NMR analysis, with deuterated dimethyl sulfoxide (DMSO) serving as the solvent. The ^1^H-NMR spectrum of [Ch][Sal] IL (Fig S3) provides valuable insights into its molecular structure. The chemical shifts of the protons are influenced by their electronic environment and the presence of neighboring groups. Protons Ha and Hb (δ 1.47 ppm) exhibit distinct chemical shifts due to the shielding effect of the oxygen atom on Ha (δ 6.87 ppm). The chemically equivalent protons H1 and H2 (δ 3.63 ppm) resonate at a similar chemical shift. In contrast, H3 and H4 (δ 3.86 ppm), also chemically equivalent, experience a deshielding effect from the adjacent carbonyl group, resulting in a downfield shift. The methyl protons (δ 1.33 ppm) associated with the choline moiety display a characteristic chemical shift. The detailed analysis of the ^1^H-NMR spectrum confirms the structural features of [Ch][Sal] IL. The observed chemical shifts for each proton type correlate with their expected positions within the molecule and the influence of neighboring functional groups.


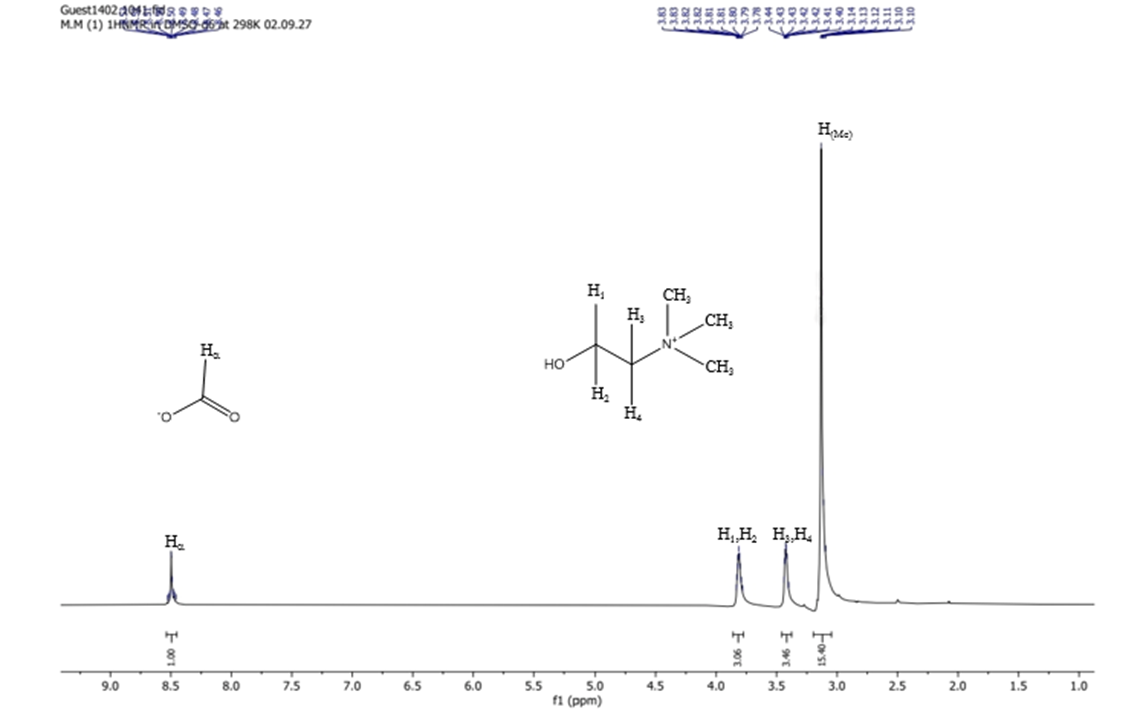


***Figure S8.*** ^1^H-NMR (400 MHz, DMSO) spectrum of [Ch][For] IL.

The ^1^H-NMR spectrum of [Ch][For] IL (Fig S6) provides valuable insights into the chemical environment of its hydrogen atoms. The aldehyde proton (Ha) experiences a significant downfield shift due to the strong electron-withdrawing effect of the adjacent carbonyl group. This places Ha at approximately 9 ppm. The methylene protons (H1 and H2) in the formate moiety resonate at around 3.81 ppm, influenced by both the electron-withdrawing carbonyl and the electron-donating oxygen. The methyl protons (H3 and H4) in the formate group exhibit a slightly lower chemical shift at 3.42 ppm, indicating less influence from the carbonyl group. Finally, the methyl protons of the choline cation appear around 3.13 ppm, shielded by the nitrogen atom.


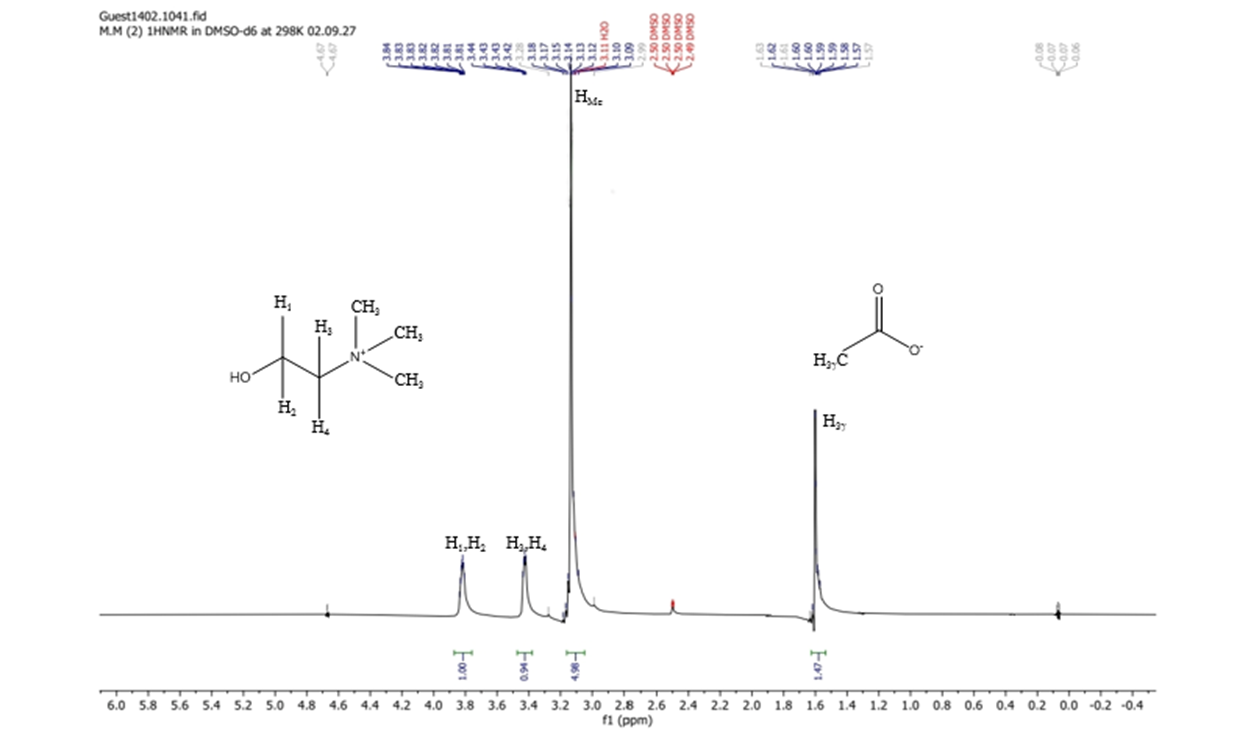


***Figure S9.*** ^1^H-NMR (400 MHz, DMSO) spectrum of [Ch][Ace] IL.

The methyl protons, influenced by the neighboring oxygen in ^1^H-NMR spectrum analysis of [Ch][Ace] IL (Fig S9), exhibit an unusually low chemical shift at approximately 1.47 ppm. In contrast, the methylene protons (H1 and H2) in the acetate moiety resonate at around 3.83 ppm, influenced by both the electron-withdrawing carbonyl group and the electron-donating oxygen. The methyl protons (H3 and H4) in the acetate group appear at a slightly higher field (3.42 ppm) compared to H1 and H2, indicating a lesser influence from the carbonyl group. Finally, the methyl protons of the choline cation resonate around 2.98 ppm, a typical chemical shift for methyl groups attached to nitrogen. The observed chemical shifts in the ^1^H-NMR spectrum of choline acetate directly correlate with the electronic environment and structural features of the molecule. The anomalous shift of the methyl protons is attributed to the shielding effect of the oxygen atom. The chemical shifts of H1, H2, H3, and H4 are influenced by the interplay between the electron-withdrawing carbonyl group and the electron-donating oxygen atom. The chemical shift of the choline methyl protons is consistent with their position relative to the nitrogen atom.

1. *Corresponding author. Tel.: +*98-41-33393094.

   Fax: +98-41-33340191.

   E-mail address: hemayatt@yahoo.com (H. Shekaari). [↑](#footnote-ref-1)
